# *In silico* prediction and biological assessment of novel angiogenesis modulators from traditional Chinese medicine

**DOI:** 10.3389/fphar.2023.1116081

**Published:** 2023-02-03

**Authors:** Yingli Zhu, Hongbin Yang, Liwen Han, Lewis H. Mervin, Layla Hosseini-Gerami, Peihai Li, Peter Wright, Maria-Anna Trapotsi, Kechun Liu, Tai-Ping Fan, Andreas Bender

**Affiliations:** ^1^ Department of Clinical Chinese Pharmacy, School of Chinese Material Medica, Beijing University of Chinese Medicine, Beijing, China; ^2^ Department of Chemistry, Center for Molecular Science Informatics, University of Cambridge, Cambridge, United Kingdom; ^3^ Department of Pharmacology, University of Cambridge, Cambridge, United Kingdom; ^4^ Engineering Research Center of Zebrafish Models for Human Diseases and Drug Screening of Shandong Province, Biology Institute, Qilu University of Technology, Shandong Academy of Sciences, Jinan, China; ^5^ School of Pharmacy and Pharmaceutical Science, Shandong First Medical University, Shandong Academy of Medical Sciences, Jinan, China

**Keywords:** TCM, angiogenesis, mode of action, machine learning, biological assessment

## Abstract

Uncontrolled angiogenesis is a common denominator underlying many deadly and debilitating diseases such as myocardial infarction, chronic wounds, cancer, and age-related macular degeneration. As the current range of FDA-approved angiogenesis-based medicines are far from meeting clinical demands, the vast reserve of natural products from traditional Chinese medicine (TCM) offers an alternative source for developing pro-angiogenic or anti-angiogenic modulators. Here, we investigated 100 traditional Chinese medicine-derived individual metabolites which had reported gene expression in MCF7 cell lines in the Gene Expression Omnibus (GSE85871). We extracted literature angiogenic activities for 51 individual metabolites, and subsequently analysed their predicted targets and differentially expressed genes to understand their mechanisms of action. The angiogenesis phenotype was used to generate decision trees for rationalising the poly-pharmacology of known angiogenesis modulators such as ferulic acid and curculigoside and validated by an *in vitro* endothelial tube formation assay and a zebrafish model of angiogenesis. Moreover, using an *in silico* model we prospectively examined the angiogenesis-modulating activities of the remaining 49 individual metabolites. *In vitro*, tetrahydropalmatine and 1 beta-hydroxyalantolactone stimulated, while cinobufotalin and isoalantolactone inhibited endothelial tube formation. *In vivo*, ginsenosides Rb3 and Rc, 1 beta-hydroxyalantolactone and surprisingly cinobufotalin, restored angiogenesis against PTK787‐induced impairment in zebrafish. In the absence of PTK787, deoxycholic acid and ursodeoxycholic acid did not affect angiogenesis. Despite some limitations, these results suggest further refinements of *in silico* prediction combined with biological assessment will be a valuable platform for accelerating the research and development of natural products from traditional Chinese medicine and understanding their mechanisms of action, and also for other traditional medicines for the prevention and treatment of angiogenic diseases.

## 1 Introduction

Angiogenesis is the physiological process of new blood vessel formation. Excessive or insufficient angiogenesis occurs in more than 70 diseases such as cancer, endometriosis, chronic wounds, and stroke (Fan et al., 2006). Excessive angiogenesis occurs when diseased cells produce abnormally large amounts of pro-angiogenic factors (e.g., vascular endothelial growth factor VEGF, and interleukin-8 IL-8) overwhelming the effects of endogenous inhibitors (e.g., angiostatin, platelet factor 4) (Ferrara and Henzel, 1989; Heidemann et al., 2003; O'Reilly et al., 1994; Bikfalvi, 2004). In these conditions, the new blood vessels feed the diseased tissues and destroy normal tissues or interfere with their functions. In contrast, inadequate blood vessel growth results in circulatory inefficiency leading to the risk of tissue death (Carmeliet, 2005).

The discoveries of endogenous pro-angiogenic and anti-angiogenic molecules and elucidation of their respective signalling pathways have led to the development of clinically effective anti-angiogenesis drugs such as monoclonal antibodies (Krasnoperov et al., 2010), small molecule tyrosine kinase inhibitors (Watanabe et al., 2021) and mTOR inhibitors (Roy et al., 2013), as well as recombinant human proteins (Jung et al., 2002). The vast reserve of natural products and herbal medicines offers a source for developing anti- or pro-angiogenic agents. For example, combretastatin and paclitaxel, derived from African bush willow tree and Pacific yew tree respectively have been developed into angiogenesis inhibitors (Wang et al., 2003; Sengupta et al., 2004). Both target microtubules and are categorized as vascular targeting agents to eradicate tumour vasculature (Pasquier et al., 2007).

In traditional Chinese medicine (TCM), many botanical drugs are used in the treatment of angiogenic diseases by modulating angiogenesis-related targets or pathways (Fan et al., 2006). However, studies have revealed that some medicinal plants can contain both pro- and anti-angiogenic phytochemicals. For example, ginseng contains ginsenosides that exert opposite effects on angiogenesis (Sengupta et al., 2004), with Rg1 having a pro-angiogenic effect (Leung et al., 2006), while Rb1 and its metabolite Rg3 have been shown to have anti-angiogenic effects (Yue et al., 2006; Leung et al., 2007). Likewise, Radix Angelica sinensis also possesses opposite angiogenesis-modulating components in its aqueous and volatile components. Aqueous extract of it promotes angiogenesis (Lam et al., 2008). In contrast, a volatile component n-butylidenephthalide derived from it inhibits angiogenesis (Yeh et al., 2011). These results highlight the importance of identifying pro- and anti-angiogenic substances in medicinal plants as well as elucidating their mechanism of action (MoA), not only for the development of novel agents for the treatment of angiogenic diseases, but also to ensure their proper and safe uses as nutraceuticals. Therefore, elucidation of MoA is of relevance to the current work, aiming to use TCM and medicinal plants from different ethnic origins as a source of angiogenesis modulators.

Ligand-based target prediction methods rely on the principle of chemical similarity, which assumes that compounds with similar chemical structure should exhibit similar biological effects (Mervin et al., 2018a; Mervin et al., 2018b; Mervin et al., 2020). While this principle generally holds across large datasets, it is not always valid, e.g., due to “Activity Cliffs,” where the activity of a compound changes abruptly, despite only minor changes in the chemical structure (Young et al., 2008; Stumpfe et al., 2019). Likewise, chemical space coverage with annotated target bioactivity information is sometimes sparse (particularly for natural products), and hence accurate prediction of targets will not be possible for a model for all chemical space, and across all protein targets.

Existing methods used to provide hypotheses for the MoA of compounds (Trapotsi et al., 2021) involve analysing chemical structures and their protein targets (Byrne and Schneider, 2019) transcriptional responses following treatment (Ravindranath et al., 2015) and text mining (Trapotsi et al., 2021). Target prediction *in silico* is a well-established computational technique capable of inferring the MoA of putative compounds by utilizing known bioactivity information (Mohamad Zobir et al., 2016). This technique is used for the deconvolution of phenotypic screens (Ehrman et al., 2007a) and has been applied to TCM by Ehrman et al. (2007b) who used Random Forest Classifier to screen 8,264 individual metabolites from 242 TCM botanical drugs *in silico*. Of particular interest, a relatively large number of botanical drugs were predicted to inhibit multiple targets, as well as the same target from different phytochemical classes (Ehrman et al., 2010; Barlow et al., 2012). Mohd Fauzi et al. reported that the MoA of the individual metabolites from the “tonifying and replenishing medicinal” class from TCM known to exhibit a hypoglycemic effect can be related to the activity of their ingredients against the sodium-glucose linked transporters (SGLT) 1 and 2 as well as protein tyrosine phosphatase (PTP).

Gene expression-based methods for the analysis of MoA (Chen et al., 2015) use the differential gene expression profile of a compound in cells upon compound perturbation. Microarrays are capable of simultaneously providing information on the expression of virtually the whole transcriptome at a time (Maggiora, 2006). In addition, more recent methods such as DRUG-Seq (Ye et al., 2018) and RASL-Seq (Simon et al., 2019) were established, which take advantage of newer sequencing technologies and thereby aim to increase the information content and decrease the cost of the data being generated. Using microarray gene expression profiling with “Connectivity Map” mining, Jiang et al. reviewed the widely applied Connectivity Map in TCM to discover the molecular mechanism and for TCM repurposing (Jiang et al., 2021). However, gene expression is only one level of information, which represents transcriptional changes in a model biological system (such as a cell line), and at a given time point and compound concentration, which may not represent the therapeutic situation *in vivo* (Bender and Cortés-Ciriano, 2021; Bender and Cortes-Ciriano, 2021). Gene expression is not regulated only by direct compound activity but is also influenced by disease processes and feedback loops, and hence while its interpretation is often not straightforward, this more complex biological setup is still closer to the reality of living systems.

Given the multi-faceted nature of MoAs, we chose to combine ligand-target predictions and gene expression data for understanding known angiogenesis modulators from TCM and constructed a Machine Learning model to predict the angiogenesis-modulating activities of unknown individual metabolites. The predictions were then validated by an *in vitro* endothelial tube formation assay (Bishop et al., 1999) and a zebrafish model of angiogenesis (Yuan et al., 2018), which we have previously used to identify individual pro-angiogenic (Yuan et al., 2018; Liao et al., 2019) or anti-angiogenic compounds (Han et al., 2012; Han et al., 2013; Li et al., 2021).

## 2 Materials and methods

### 2.1 Materials

#### 2.1.1 Individual metabolites from TCM for biological assessment

Curculigostide (PHL80576, ≥98%), Deoxycholic Acid (D2510, ≥98%) and Tetrahydropalmatine (SMB00339, ≥98%) were purchased from Sigma-Aldrich, Gillingham, Dorset, United Kingdom. Ferulic acid (19,871, ≥98%), Ursodeoxycholic Acid (21,892, ≥98%), Isoalantolactone (33,838, ≥98%) Cinobufotalin (19,845, ≥98%) and Ginsenosides Rg1 (15,315, ≥98%), Ginsenosides Rb3 (29,005, ≥98%) and Ginsenosides Rc (29,088, ≥98%) were purchased from Cayman Chemical, Ann Arbor, Michigan, United States. 1 beta-hydroxyalantolactone (RB17897, ≥95%) were purchased from Bioruler, Connecticut, United States.

#### 2.1.2 *In vitro* and *in vivo* materials

Human umbilical vein endothelial cells (HUVECs) and human dermal fibroblasts (HDFs) were purchased from PromoCell Cells (Heidelberg, Germany) and were subsequently cultured in PromoCell’s Endothelial Cell Growth Medium 2 (EGM-2) containing 2% foetal bovine serum (FBS), human epidermal growth factor (EGF), human basic fibroblast growth factor (bFGF), insulin-like growth factor (IGF), human vascular endothelial growth factor (VEGF), ascorbic acid and heparin. Endothelial Cell Growth Medium-2 as well as their respective bullet kits were purchased from PromoCell. Trypsin 0.005%/EDTA 0.01% solution, Dulbecco A Phosphate Buffered Saline (PBS), 1-StepTM NBT/BCIP (Pierce Protein Research) were purchased from Thermo Scientific, Loughborough, United Kingdom. Foetal bovine serum (FBS), dimethyl sulfoxide (DMSO), paraformaldehyde, Triton^®^ X-100, rabbit anti-human von Willebrand factor antibody (F-3520), and mouse anti-rabbit IgG-alkaline phosphatase (A9919) were purchased from Sigma-Aldrich, Gillingham, Dorset, United Kingdom. Human recombinant VEGF and precast gels were purchased from Invitrogen Life Technologies, Paisley, United Kingdom. PTK787 (vatalanib dihydrochloride) was purchased from MedChem Express, Monmouth Junction, United States. Pronase E were purchased from Solarbio, Shanghai, China.

### 2.2 Experiment design

**FIGURE 1:**
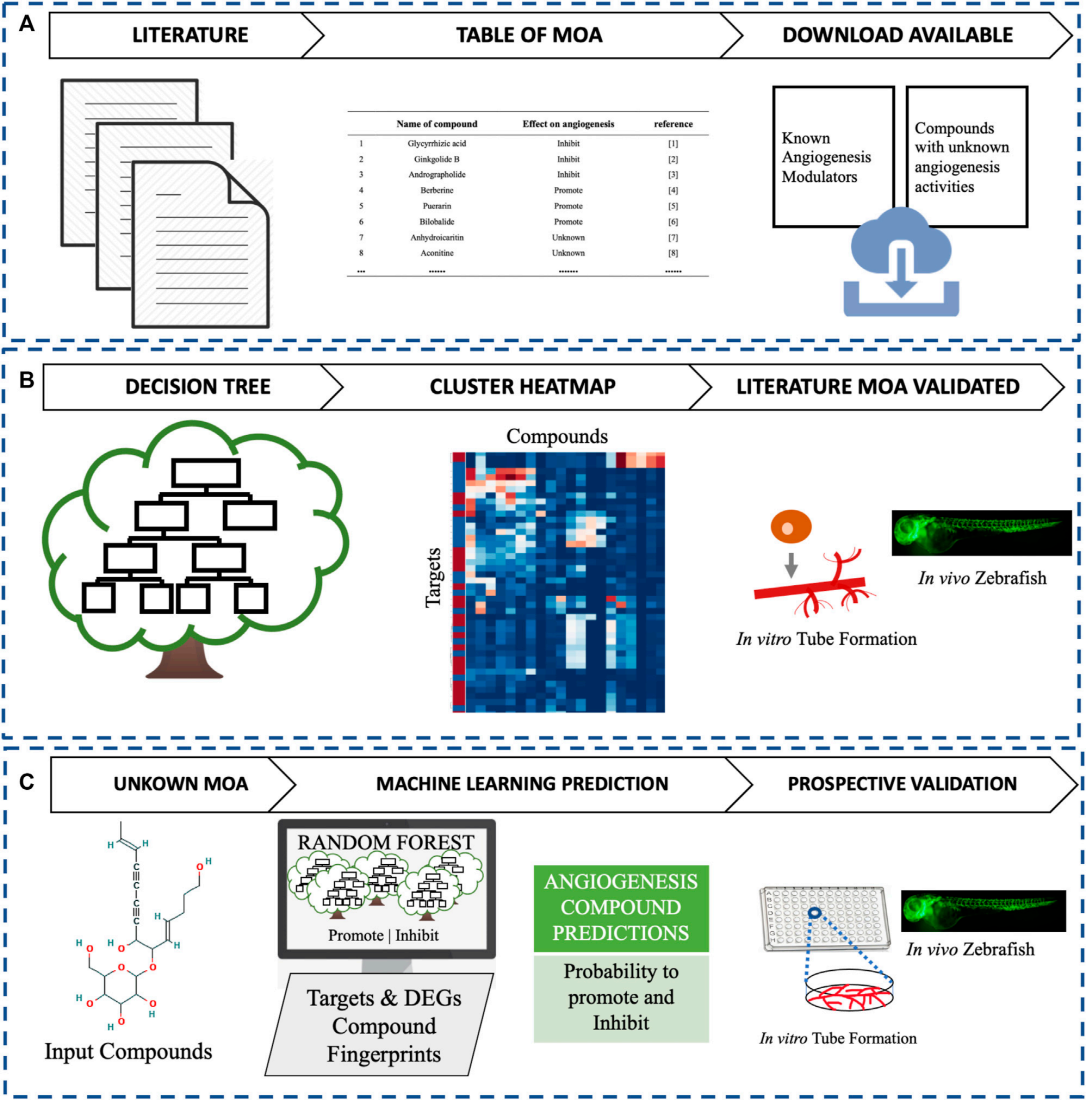
This study compromises three main parts. **(A)** Literature assessment of angiogenesis-modulating individual metabolites from TCM, **(B)** Decision tree and clustering analysis to validate known mechanisms, and **(C)** Identification and assessment of new small-molecule modulators of angiogenesis *in vitro* and *in vivo*.

In this study, as shown in Figure 1, we have performed a literature search to build a database of TCM individual metabolites that promote or inhibit angiogenesis. Then, we annotated the individual metabolites with their predicted targets and experimentally measured Differentially Expressed Genes (DEGs) to rationalize the MoA of the angiogenesis phenotype and with decision trees aimed to rationalize the poly-pharmacology of the angiogenesis phenotype. We subsequently created a Machine Learning model, trained on both DEGs and protein targets to predict a compound’s ability to modulate angiogenesis. Finally, the predictions were assessed by an *in vitro* endothelial tube formation assay (Bishop et al., 1999; Liao et al., 2019) and an *in vivo* zebrafish model (Han et al., 2012; Han et al., 2013).

### 2.3 Compound set preparation

The compound data set used for this work comprised 100 active TCM individual metabolites as published in previous work (Lv et al., 2017), which were commonly found in TCM botanical drugs, such as *Salvia miltiorrhiza* Bunge [Lamiaceae; Salviae miltiorrhizae radix et rhizoma], *Coptis chinensis* Franch. [Ranunculaceae, Coptidis Rhizoma], and *Panax ginseng* C.A.Mey [Araliaceae, Ginseng Radix et Rhizama]. These were downloaded from the Gene Expression Omnibus (GEO) using the accession number GSE85871 (the full list of individual metabolites with annotations is given in Supplementary Table S1). Most of these 100 individual metabolites are reported to be quality-controlled components in the Chinese Pharmacopoeia and have been selected to represent a wide range of activities and diverse structures (Lv et al., 2017). According to our literature review, 51 of the 100 active TCM individual metabolites studied were known to modulate angiogenesis (Known Angiogenesis Modulators), while the ability of the remaining 49 individual metabolitesto modulate angiogenesis was unknown to date. Of the 51 Known Angiogenesis Modulators 19 were known promoters and 32 were known inhibitors. The gene expression data obtained from GEO used all individual metabolites at either 1 or 10 μM, and DMSO used as control, tested on MCF7 breast cancer cell lines in duplicate. Total RNA was extracted and profiled by Affymetrix HG U133 A 2.0 microarray chips. Compound structures, represented by Simplified Molecular Input Line Entry System (SMILES), were pre-processed using the open access eTOX standardiser (https://github.com/flatkinson/standardiser), with the options set to “aromatize,” and “keep largest fragment.”

### 2.4 *In silico* target prediction and enrichment calculation for known angiogenesis modulators

Prediction IncluDinG INactivity (PIDGIN) (version 3) was used to conduct target prediction for the 100 individal metabolites mentioned above, by which the probability of the association between a compound and a drug target could be predicted. We used the following function options: bioactivity threshold is 10 μM. A background of 4,041 TCM molecules were selected from the TCM database at TCM@TAIWAN and SuperTCM (Chen et al., 2021). The individual metabolites were pre-processed using the same protocol above. We then used this set of TCM molecules as a background TCM chemical space reference set (putative inactive individual metabolites for the angiogenesis phenotype), which was needed subsequently to calculate enrichment for the targets more often linked to the promoter or inhibitor phenotype (Mervin et al., 2018a). In brief, the Fishers Exact *t*-Test is performed on the contingency table for the number of active and inactive target predictions in the promoter or inhibitor set of individual metabolites (a) and (b), respectively, compared to the number of active and inactive predictions in the background (putative inactive) TCM set, (c) and (d), respectively. The output of the enrichment calculation is the Odds Ratio (OR), defined as:

$$OR=\frac{a/\left(a+b\right)}{c/\left(c+d\right)}$$ (1)

An OR score over 1.0 represents enrichment for a target in the promoter or inhibitor set (i.e., a target of possible relevance for angiogenesis modulation), whilst a score below 1.0 represents enrichment in the reference TCM set (i.e., the target is less likely to be associated with angiogenesis, based on the data analyzed). A Fishers Exact *p*-value is generated, where a value below 0.05 indicates when enrichment can be considered significant, and thus where we may reject the null hypothesis (that there is no difference in the target predictions between either promoter or inhibitor set and a background of 4,041 TCM reference individual metabolites). Given the biases in chemical space the application of statistical tests needs to be interpreted with care; however, the above framework provides an empirical way to prioritize proteins more (and less) likely involved in a particular MoA, given a set of ligand-target interactions in two datasets. Target Prediction enrichment profile hierarchical clustering analysis for MoA hypothesis generation was then conducted using Seaborn Clustermap (version 0.11).

### 2.5 Gene expression analysis for known angiogenesis modulators

Raw CEL data downloaded from GEO were first normalized by Robust Multiarray Average (Bolstad et al., 2003) using Affymetrix Power Tools. For data analysis, we collapsed the probe sets representing the same gene using the maximum expression value of these probe sets. Gene expression values from the duplicates were averaged, and a fold change of 1.5 was used as the thresholds to select DEGs for each TCM component against DMSO. We used the list of DEGs as the gene signatures for subsequent bioinformatics analysis.

### 2.6 Pathway enrichment analysis for known angiogenesis modulators

We used Over-Representation Analysis (ORA) (Karp et al., 2021) implemented in clusterProfiler (version 2.1.0) (Yu et al., 2012), an R package, to perform the analysis of TCM component treatment versus DMSO gene expression profiles. We used the KEGG gene sets (https://www.kegg.jp/kegg/) in the analysis and performed pathway enrichment using limma (version 3.38.3) (Ritchie et al., 2015). As this is discovery research of gene sets/pathways for TCM Mechanism of Action (MoA), we considered pathways/gene sets with *p* < 0.05 (adjusted for multiple testing) as significantly enriched. We kept only pathways which have significant enrichment (adjusted *p* ≤ 0.05) in two or more TCM individual metabolites for visualization. Pathway enrichment profile hierarchical clustering analysis for MoA hypothesis generation was then conducted using Seaborn Clustermap (version 0.11).

### 2.7 Decision tree generation for visualising important target proteins and differentially expressed genes

We next trained a decision tree implemented in Scikit-learn (version 0.22) (Abraham et al., 2014) to merge both predicted protein targets of ligands, as well as dysregulated genes in the form of DEGs after compound application, to arrive at a joint target protein/gene mode of action profile of angiogenesis modulators. Both types of information are of very different nature, but they are complementary in the way they represent compound action (and the suitability of this type of integrated mode-of-action representation was one of the aspects of the work we aimed to explore here). To this end, the Scikit-learn Decision Tree Classifier was used with the max_depth set to 20, the min_samples_leaf set to 4, the splitting criterion set to entropy and the class weights set to balanced. The tree was trained on the exhaustive combinations of target prediction and DEG profiles across the 100 individual metabolites, whilst supplying the sample_weight parameter with the weights of the inhibit and promote individual metabolites from the class_weight.compute_class_weight.sample_weight function, to correct for the imbalance between promoting and inhibiting individual metabolites. The classification decision tree was finally visualised using the plot_tree function.

### 2.8 Training a predictive angiogenesis random forest model and prospective prediction for unknown angiogenesis modulators

A Random Forest classifier as implemented in Scikit-learn (version 0.22) was trained. Random Forest Classifier (Ravindranath et al., 2015) function with the “n_estimators” set to “2000” the “class_weight” parameter set to “balanced.” The model was trained on the set of features present and absent from either target prediction and/or DEG. The output of the algorithm is the likelihood ranging from 0.0 to 1.0 that an input compound will promote or inhibit angiogenesis, respectively. We used leave-one-out cross validation (CV) to evaluate the model performance. Considering the imbalanced training data between angiogenesis promotors and inhibitors, we shifted the probability threshold from 0.5 to 0.4 according to the CV results, which means less individual metabolites would be predicted to be promotors. Tree-based machine learning algorithms were used because these methods provided better interpretability and the importance of the features could be obtained (Breiman, 2001). By analyzing the different decision tree models in the random forest model, we found that most trees used less than 10 features. Therefore, we only select the best 10 targets or DEGs to build the explainable models. As for the final models that were used to predict the unknown TCM individual metabolites, Random Forest model was choosen due its high predictive performance and less risk of over-fitting relative to a single decision tree.

### 2.9 Identification of novel angiogenic TCM metabolites

To test the general applicability of the above Machine Learning model, we used an *in vitro* tube formation assay (Choi et al., 2021) and *in vivo* zebrafish model (Liao et al., 2019) to validate the predicted novel individual angiogenic metabolites as described in detail in the following section.

#### 2.9.1 *In vitro* endothelial tube formation assay

In the 12‐days co‐culture model, when human fibroblasts are co‐cultured with endothelial cells, the fibroblasts secrete the necessary matrix components that act as a scaffold for tube formation (Bishop et al., 1999). In contrast to the 6 h Matrigel assay, this 12‐days assay has been shown to produce tubes that contain lumen, and a more heterogeneous pattern of tube lengths that more closely resemble capillary beds *in vivo* (Karp et al., 2021). Briefly, HUVECs and HDFs were cultured in EGM-2, until sub-confluent. Cells were then seeded in 96-well flat-bottom plates at a 1:20 ratio cells per well, cultured in EGM-2 medium. After 2 days, media was replenished, and on day 4, EGM-2 was titrated 10-fold with its basal medium (EBM-2) and 2% FBS. Individual Metabolites treatment in new medium were replenished every 2 days before staining on day 12. At Day 12, cells were fixed with 10% formalin, incubated with a rabbit anti‐human‐von Willebrand factor (vWF) monoclonal IgG (1:1,000; Sigma‐Aldrich, Gillingham, United Kingdom; Cat# F3520), and then a mouse anti‐rabbit alkaline phosphatase conjugated IgG (1:1,000; Sigma‐Aldrich; Cat# A9919). Following washes, one‐Step TM NBT/BCIP kit (Thermo Scientific, Loughborough, United Kingdom) was applied until a suitable signal was developed. Images of two fields of view were taken per well. The total tube area and average tube size of vWF‐positive endothelial cells forming capillary‐like tubes were quantified by the ImageJ software (https://imagej.nih.gov/ij/).

#### 2.9.2 *In vivo* zebrafish assays

Transgenic zebrafish (Tg [vegfr2: GFP]) were maintained in 3 L polystyrene aquarium tanks with constant aeration and flow water systems at 28°C ± 1°C under a 14‐hr light/10‐hr dark photoperiod. Food with brine shrimp was fed twice per day. For breeding, adult zebrafish were placed in 1.5 L breeding tanks overnight and were separated by a transparent barrier that was removed on the following morning. Zebrafish embryos were raised in culture water (containing 5.00 mM NaCl, 0.17 mM KCl, 0.44 mM CaCl_2_, 0.16 mM MgSO_4_). Healthy, hatched zebrafish embryos were picked out and staged by time and morphological criteria (Cannon, Upton, Smith, and Morrell, 2010). Randomization was used to assign embryos to different experimental groups and to the drug treatment.

In the experiments for testing anti-angiogenic activities (Han et al., 2012; Han et al., 2013), the test individual metabolites (deoxycholic acid and ursodeoxycholic acid) were dissolved in DMSO and then further diluted in culture water to the required concentrations. The zebrafish embryos of test groups were treated with test compound samples, without PTK787. Positive control embryos were treated with 0.2 μg/mL PTK787. Other procedures were the same as in the pro-angiogenic assay.

In pro-angiogenic experiments (Yuan et al., 2018; Liao et al., 2019), the individual metabolites selected for *in vivo* assessment, PTK787, ginsenoside Rg1, Ferulic acid, Curculigoside, Tetrahydropalmatine, 1-Beta-Hydroxyalantolactone, Cinobufotalin, Isoalantolactone, Ginsenosides Rb3 and Rc were dissolved in DMSO and then further diluted in culture water to the required concentrations. 24 h post‐fertilization (hpf) embryos were dechorionated with 1 mg/mL of Pronase E before treatment. Control embryos were treated with the equivalent amount of DMSO solution (final concentration: 0.1% DMSO [v/v]). Model embryos were treated with 0.2 μg/mL PTK787. Positive control embryos were treated with 0.2 μg/mL PTK787 and 40 μM ginsenoside Rg1. The zebrafish embryos of test groups were treated with 0.2 μg/mL PTK787 and test compound samples. After 24 h of incubation at 28.0°C ± 1°C with a 14‐hour light/10‐hour dark cycle, embryos were anaesthetized with 0.02% tricaine methane sulfonate and photographed under a fluorescence microscope (Olympus SZX16, Tokyo, Japan). The length of intersegmental vessels (ISVs) between the trunk and tail of each embryo was measured with the Image Pro Plus 5.0 by a user blinded to the exposure groups.

The experiment procedures were conducted according to the standard ethical guidelines that were approved by the Ethics Committee of the Biology Institute of Shangdong Academy of Science (SWS20210306).

### 2.10 Statistical analysis

All in tube formation assay data were rendered as means ± SEM and the statistical results were analysed by a one-way ANOVA in SPASS 20.0 (https://www.ibm.com/products/spss-statistics). *p*-Values below 0.05 were considered as statistically significant. All zebrafish data were processed by GraphPad Prism 6.0 software (https://www.graphpad.com/). After statistical analysis data were shown as mean ± SEM. The comparison between groups was performed by student’s test. *p*-values below 0.05 were considered as statistically significant.

## 3 Results

### 3.1 Enriched prediction targets for known angiogenesis modulators

First of all, the 51 individual TCM metabolites with known angiogenesis activities were subjected to bioactivity prediction and the subsequent calculation of enrichment metrics when compared to 4,041 TCM compound predictions. Enrichment analysis was performed to select significant proteins, which are more likely to be related to the mode of actions of angiogenesis. Table 1 shows the predictive bioactivities between the 51 TCM individual metabolites and the 20 proteins with high enrichment factors (odds ratio’s) and low *p*-values. It can be seen that these proteins including CYP450 2C19, Maltase-glucoamylase, Galectin-9, Microtubule-associated protein tau and Carbonic anhydrase IV are significantly related to angiogenesis promotion with enrichment factors ranging from 2.93 to 8.18 and Fisher’s Test *p*-values below 0.03. These are consistent with our literature review. For example, Macrophage migration inhibitory factor (Shimizu et al., 1999; Ogawa et al., 2000) and P-selectin (Egami et al., 2006) frequently play an essential role in the formation of new blood vessels.

**TABLE 1:** Ten most enriched predicted protein targets in either pro-angiogenic or anti-angiogenic metabolites from TCM. Predicted protein targets selected and ranked by Fisher’s exact test *p* values.

	Uniprot	Name	Fishers test *p*-value	Odds ratio
PROMOTE	P33261	Cytochrome P450 2C19	0.00791	4.84
	O43451	Maltase-glucoamylase	0.00943	4.05
	O00182	Galectin-9	0.0113	8.18
	P10636	Microtubule-associated protein tau	0.0144	4.13
	P22748	Carbonic anhydrase IV	0.0187	2.93
	P16109	P-selectin	0.0234	4.24
	P14174	Macrophage migration inhibitory factor	0.0355	2.91
	P17931	Galectin-3	0.0421	3.35
	P30305	Dual specificity phosphatase Cdc25B	0.0423	3.03
	P05067	Beta amyloid A4 protein	0.0464	2.95
INHIBIT	P54707	Potassium-transporting ATPase alpha chain 2	0.00122	55.1
	P80365	11-beta-hydroxysteroid dehydrogenase 2	0.00206	3.77
	P07478	Trypsin II	0.00209	39.2
	P36873	Serine/threonine protein phosphatase *p*P1-gamma catalytic subunit	0.00247	12.9
	Q8TDU6	G-protein coupled bile acid receptor 1	0.00385	5.45
	Q14973	Bile acid transporter	0.00482	23.5
	Q12908	Ileal bile acid transporter	0.00482	23.5
	P11473	Vitamin D receptor	0.0120	13.8
	P28845	11-beta-hydroxysteroid dehydrogenase 1	0.0146	12.3
	P14410	Sucrase-isomaltase	0.0493	3.14

**FIGURE 2:**
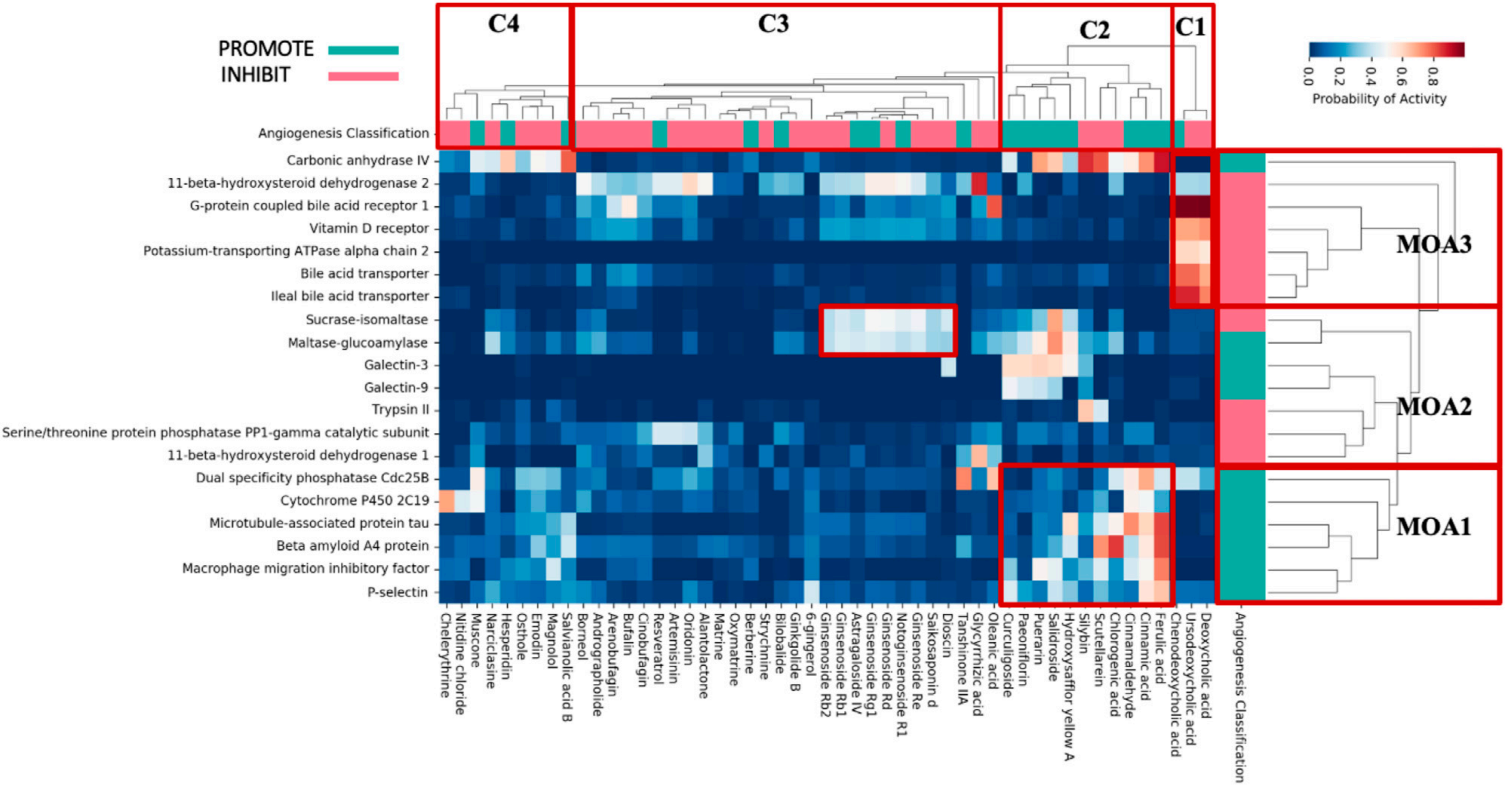
Protein bioactivities predicted by PIDGIN between individual TCM metabolites and most enriched targets. Individual metabolites were clustered based on the profile of target prediction (C1–C4); pink and green represent literature evidence for inhibition (INHIBIT) and promotion (PROMOTE) of angiogenesis, respectively, whilst the enriched anti-angiogenic (INHIBIT) and pro-angiogenic (PROMOTE) protein targets are shown in the MoA column on the right, respectively.

Hierarchical clustering was next conducted to further understand the potential mode of actions of these 51 TCM metabolites, resulting in three mode-of-action classes (MoA1-MoA3) and four clusters of metabolites (C1-C4), shown in Figure 2.

Six targets are clustered in MoA1 of which metabolites in cluster 2 observed high predicted bioactivities. This indicates that the angiogenic activity of these metabolites may be a result their interaction with this set of proteins. For example, ferulic acid has been reported to stimulate or inhibit angiogenesis depending on the experimental model used. For example, it has been reported to augment angiogenesis, both *in vitro* and *in vivo*, through the modulation of VEGF, platelet-derived growth factor (PDGF) and hypoxia-inducible factor-1 alpha (HIF-1α) (Lin et al., 2010). However, other studies showed that ferulic acid inhibits endothelial cell proliferation through nitric oxide (NO), and by downregulating the extracellular-regulated protein kinases1/2 (ERK1/2) pathway (Hou et al., 2004). More recently, Yang et al. (2015) showed that ferulic acid targets the FGFR1-mediated PI3K-Akt signaling pathway, leading to the suppression of melanoma growth and angiogenesis. Overall, we observe that the protein target prediction profiles in MoA1 are significantly enriched with metabolites that inhibit/promote angiogenesis and have been traditionally used in the modulation of angiogenesis.

Of the targets in MoA2 there is a pronounced pattern in compound cluster 2, where all the five constituent metabolites (Hydroxysafflor yellow A, Salidroside, Puerarin, Paeoniflorin, Curculigoside) have high predicted bioactivities for Galectin-3 and Galectin-9 both of which have been shown to be relevant to angiogenesis. Galectin-3 protects against ischemic stroke by promoting neuro-angiogenesis (Wesley et al., 2021). While Galectin-9 is a mammalian lectin secreted by endothelial cells and induces the phosphorylation of Erk 1/2, p38, and JNK to mediate angiogenesis (O'Brien et al., 2018). The targets involved in angiogenesis are likely to be proteins that influence either their production or associated signalling (Heng et al., 2013).

Seven targets are clustered in MoA3 of which the three bile acid metabolites in cluster 1 (Deoxycholic acid, Ursodeoxycholic acid, Chenodeoxycholic acid) observed high predicted bioactivities. The targets present in MoA3 have known association with angiogenesis. For example, G-protein coupled bile acid receptor 1 (GPBAR) can influence angiogenesis through suppressing the proinflammatory cytokine production and phagocytic function of macrophages, and by enhancing the barrier function *via* cAMP/protein kinase A (pkA)/Rac1-dependent signal pathway (Chung et al., 2009; Kida et al., 2014). Next, the Vitamin D receptor can stimulate the proliferation and development of capillary-like tubules of endothelial colony-forming cells, and has been shown to inhibit developmental angiogenesis in the zebrafish larval eyes leading to abnormal tumor angiogenesis (Merrigan and Kennedy, 2017). Finally, the bile acid transporter and the ileal bile acid transporter both play an important role in sodium-dependent reabsorption of bile acids from the lumen of the small intestine (Alam et al., 2014).

Bile acids themselves have been shown to induce overexpression of homeobox gene CDX-2 and vascular endothelial growth factor (VEGF). Studies have also demonstrated that bile acids can induce endothelial dysfunction by enhancing expression of intercellular adhesion molecule 1 (ICAM-1), vascular cell adhesion protein 1 (VCAM-1), and E-selectin *via* stimulation of the NF-κB (nuclear factor kappa of activated B cells) and p38 MAPK pathways (Soma et al., 2006). Hydrophilic bile acids such as Chenodeoxycholic acid plays a potential role in hepatic tissue regeneration by enhancing angiogenesis, whereas at higher concentrations, hydrophobic bile acids could lead to vascular damage (Zhao and Adjei, 2015).

### 3.2 Analysis of gene expression enrichment for known angiogenesis modulators

**FIGURE 3:**
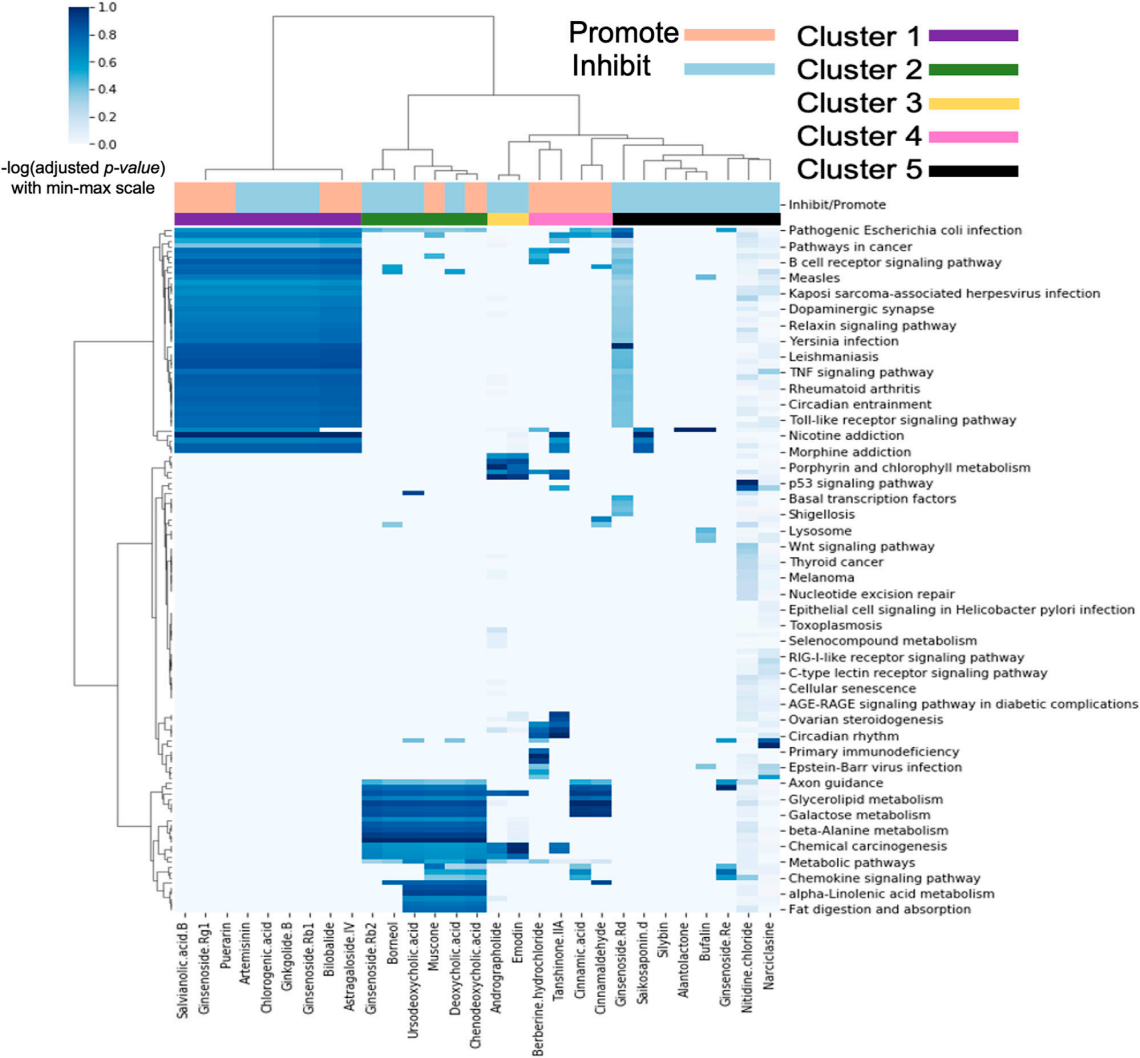
Pathway enrichment signatures for individual pro-angiogenic (Promote) or anti-angiogenic (Inhibit) metabolites derived from TCM. Individual metabolites was clustered into five groups labelled with different colours (Cluster 1–5).

We next analysed the biological pathway(s) transcriptionally modulated after administration of TCM metabolite in MCF7 cells using Over-Representation Analysis (ORA). Figure 3 shows the heatmap of enriched pathways for individual TCM metabolites using KEGG gene sets as described in Materials and Methods.

The heatmap shows 5 MoA clusters; Cluster 1 and 2 have very well-defined similarities in pathway activity whereas Clusters 3, 4, and 5 are smaller and less well-defined, although we were still able to find literature evidence for inhibiting or promoting angiogenesis.

Cluster 1 metabolites have activity toward a range of angiogenesis pathways especially related to cancer and the immune system. Firstly, the B cell receptor signalling pathway which describes the role of B cells in immunity. The role of B cells in tumour initiation, progression, and angiogenesis is still debated. However, there is clinical evidence regarding their association with good prognosis of cancer patients and potential anti-tumour effect (Naserian et al., 2020). Secondly, the TNF signalling pathway is particularly relevant as TNF-alpha is known to drive remodelling of blood vessels and has multiple roles in angiogenesis, which is known to be related to cancer and immunity (Hu et al., 2019). Next, the Toll-like receptor signalling pathway encompasses signalling of various Toll-like receptors which play a key role in the immune system (Xu et al., 2013), and Toll-like receptor 2 and 4 have been found to induce angiogenesis (Sun et al., 2016; Potente and Carmeliet, 2017). Finally, the Relaxin signaling pathway was identified. Relaxin is a hormone which stimulates angiogenesis by up-regulating VEGF (Neschadim et al., 2015).

The pathways enriched in metabolites cluster 2 are related to the metabolism of various chemicals including glycerolipid, galactose, beta-alanine and alpha-linolenic acid. Angiogenesis has traditionally been viewed from the perspective of how endothelial cells (ECs) coordinate migration and proliferation in response to growth factor activation to form new vessel branches (Shimo et al., 1999). However, ECs must also coordinate their metabolism and adapt metabolic fluxes to the rising energy and biomass demands of branching vessels. Recent studies have highlighted the importance of such metabolic regulation in the endothelium and uncovered core metabolic pathways and mechanisms of regulation that drive the angiogenic process (Moccia et al., 2019). Additionally, this cluster contains the Chemokine signalling pathway. Chemokines, a large family of inflammatory cytokines, have been shown to play a critical role in the regulation of angiogenesis during several pathophysiologic processes, such as tumour growth, wound healing, and ischemia (Pozzobon et al., 2016).

As previously mentioned, clusters 3, 4, and 5 are less well-defined but nevertheless show pathway activity for several processes relevant to angiogenesis. This includes the p53 signalling pathway which modulates angiogenesis in multiple ways, such as *via* promotion of VEGF expression (Farhang Ghahremani et al., 2013), inhibition of proangiogenic factors (Pfaff et al., 2018) and interference of central regulators of hypoxia that mediate angiogenesis (Sethi et al., 2019). Also enriched is the Wnt signalling pathway, which has been linked to proper vascular growth in murine and human retina (Wang et al., 2019). Additionally, the angiogenic factor Norrin acts through the Wnt receptor, Frizzled 4 (Sethi et al., 2019).

Overall, this analysis identified distinct pathways modulated by individual metabolites with angiogenic activity based on the transcriptional changes they induce. This also implies that these signatures can be used to predict whether an individual TCM metabolite can promote or inhibit angiogenesis.

### 3.3 Explanation of the machine learning model

**FIGURE 4:**
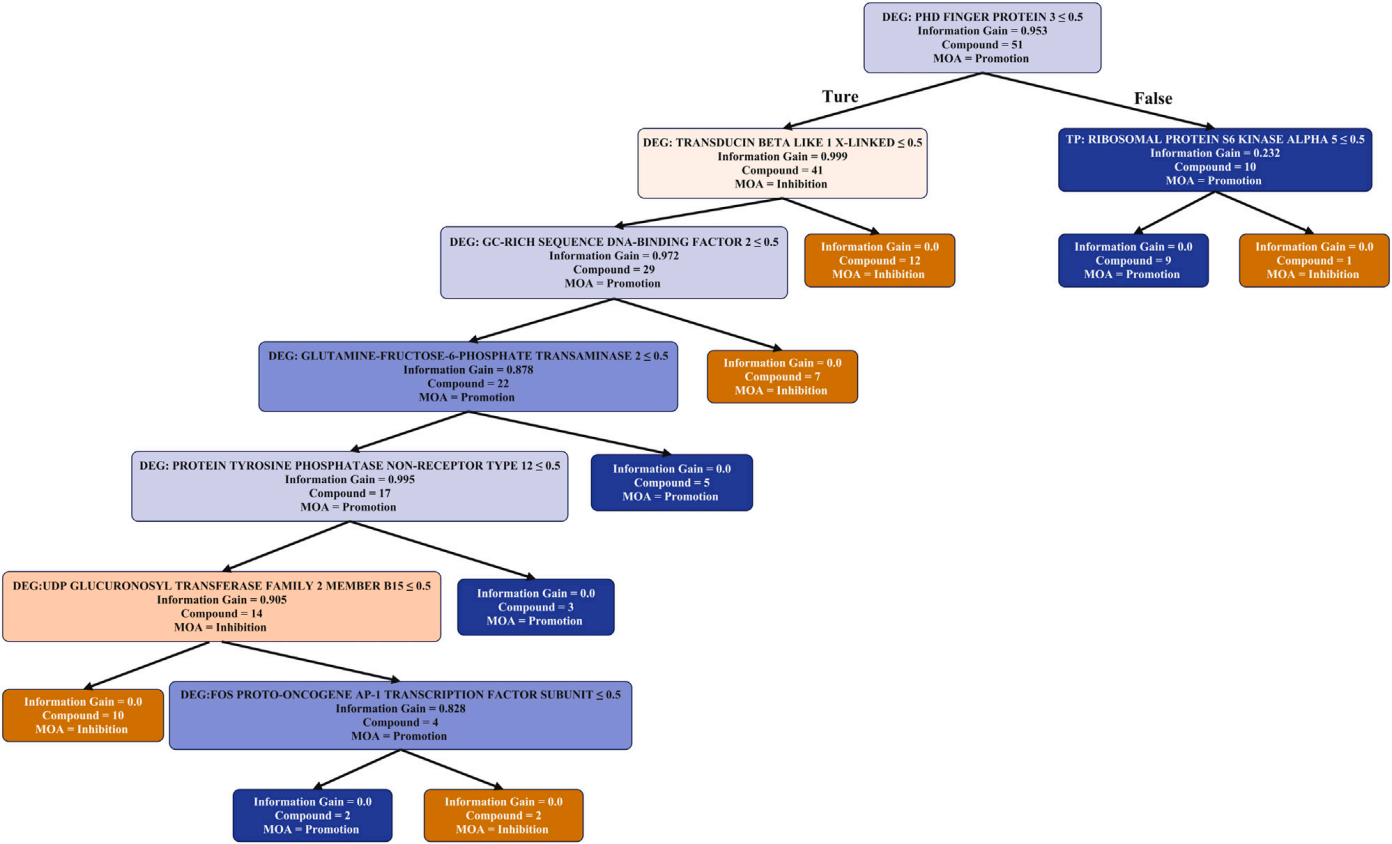
Mode of action analysis of individual pro- and anti-angiogenic metabolites derived from TCM, based on the most important ten predicted targets and DEGs. The number of metabolites in each node is shown, along with the entropy (the information gain) to denote the quality of each split in the tree. Colour is used to indicate the purity for PROMOTER (blue) or INHIBITOR (orange) classification of metabolites. It can be seen that both DEGs and Target prediction were selected by the decision tree as important features that can distinguish between inhibitors and promoters.

To gain further insight into the MoA of angiogenesis modulators we next generated decision tree and Random Forest classification models to predict angiogenic activity. Decision trees were utilised due to their interpretability and visualization (Figure 4), and Random Forests were used due to their enhanced predictive power. To this end, three sets of descriptors were used: *in silico* protein bioactivity predictions (Supplementary Figure S1), Differentially Expressed Genes (DEGs; Supplementary Figure S2), and a combination thereof (Figure 4) based on the ten most important features from either input space ranked by Gini importance (details shown in Supplementary Table S2).

In the Random Forest model utilising protein bioactivities Ribosomal protein S6 kinase alpha 5 (RPS6KA5) had the highest importance score by Gini importance (Supplementary Table S1). This protein was also at the top level in the decision tree built with protein bioactivities with seven known angiogenesis promoters being predicted as active. RPS6KA5 may be related to inhibition of angiogenesis, which is involved in the MAPK signalling pathways (Dong et al., 2019) to suppress angiogenesis (Yang et al., 2018). Endoplasmic reticulum aminopeptidase 1, which is listed as the second most important target in Supplementary Table S2, may also be related to angiogenesis because the 5 individual metabolites which were predicted to be active against this protein are known angiogenesis promoters (Cifaldi et al., 2012). On the other hand, sodium/glucose cotransporter 2 (Kaji et al., 2018) may be related the promotion of angiogenesis, because three individual metabolites that were predicted to be active against this protein are known angiogenesis promoters. Therefore, we can conclude that the machine learning models using target prediction descriptors is able to identify important target proteins related to angiogenesis.

From the decision tree using both TP and DEGs descriptors (Figure 4), it can be seen that the most important gene was PHD Finger protein located at the top of the decision tree. For the 10 individual metabolites which can regulate the expression level of this gene, 9 are promoters and only 1 is an inhibitor. It could be further recognized by the protein activity of Ribosomal protein S6 kinase alpha 5, the most important protein in the TP-only decision tree. For the remaining individual metabolites, their regulation of either “Transducin beta like 1 X-linked” or “GC-rich sequence DNA-binding factor 2” indicates the inhibition of angiogenesis. From this analysis we can conclude that these DEGs play an important role in the model as most of the nodes in the TP and DEG decision tree using both are DEGs.

### 3.4 Biological assessment of known angiogenesis metabolites

**FIGURE 5:**
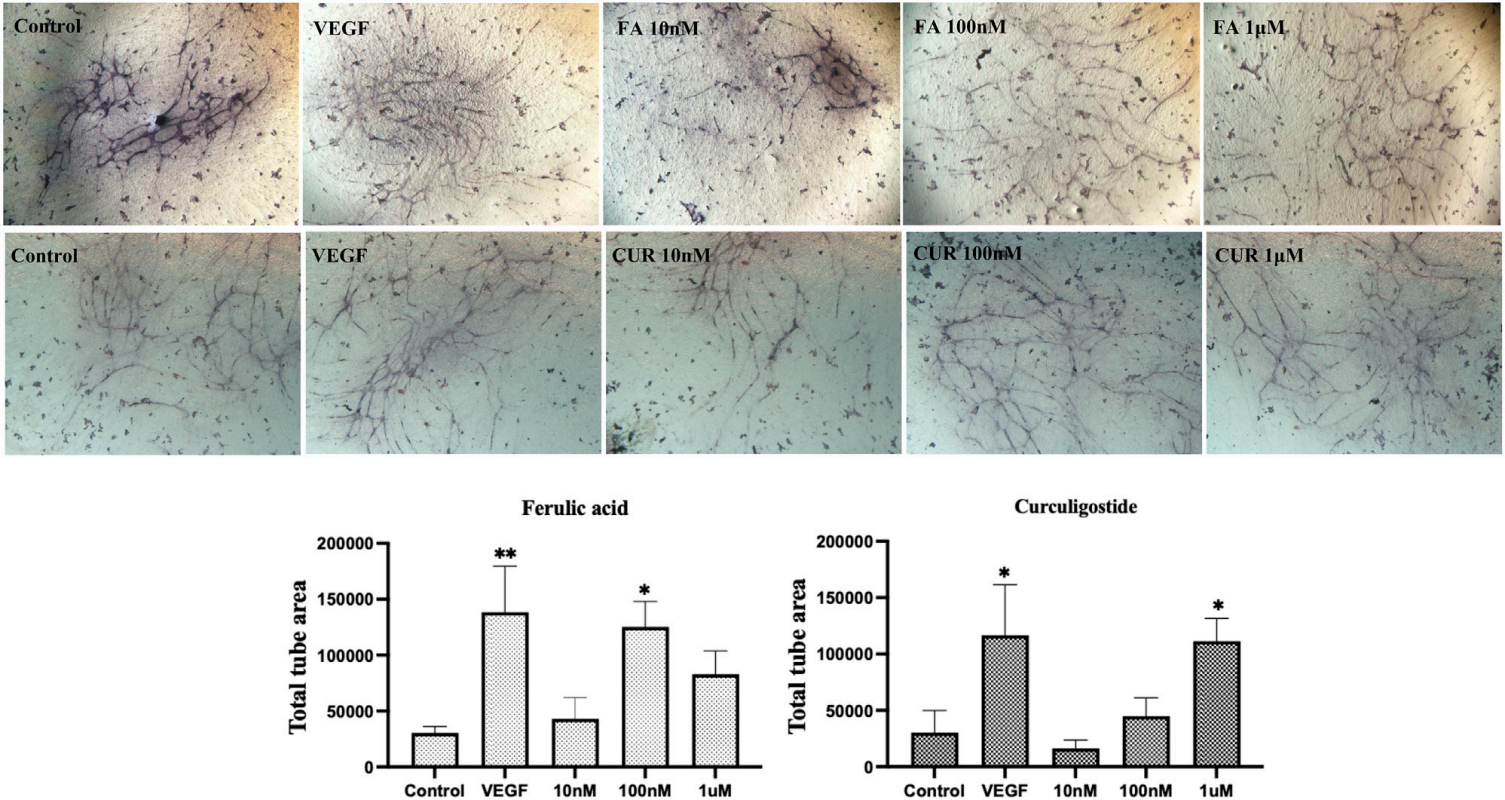
Modulation of endothelial tube formation by ferulic acid (FA) and curculigostide (CUR) *in vitro*. In a HUVEC-HDF co-culture model, ferulic acid stimulated endothelial tube formation at 10 nM but produced an inhibitory effect at higher concentrations. Curculigostide stimulated endothelial tube formation at 10 nM–1 μM. VEGF was included as the positive control. Statistical analyses were performed by one‐way ANOVA followed by Dunnett’s *post hoc* test. Data represents mean ± SEM (*n* = 4–6). Compared with the control group, *p < 0.05, **p < 0.01, ***p < 0.001.

**FIGURE 6:**
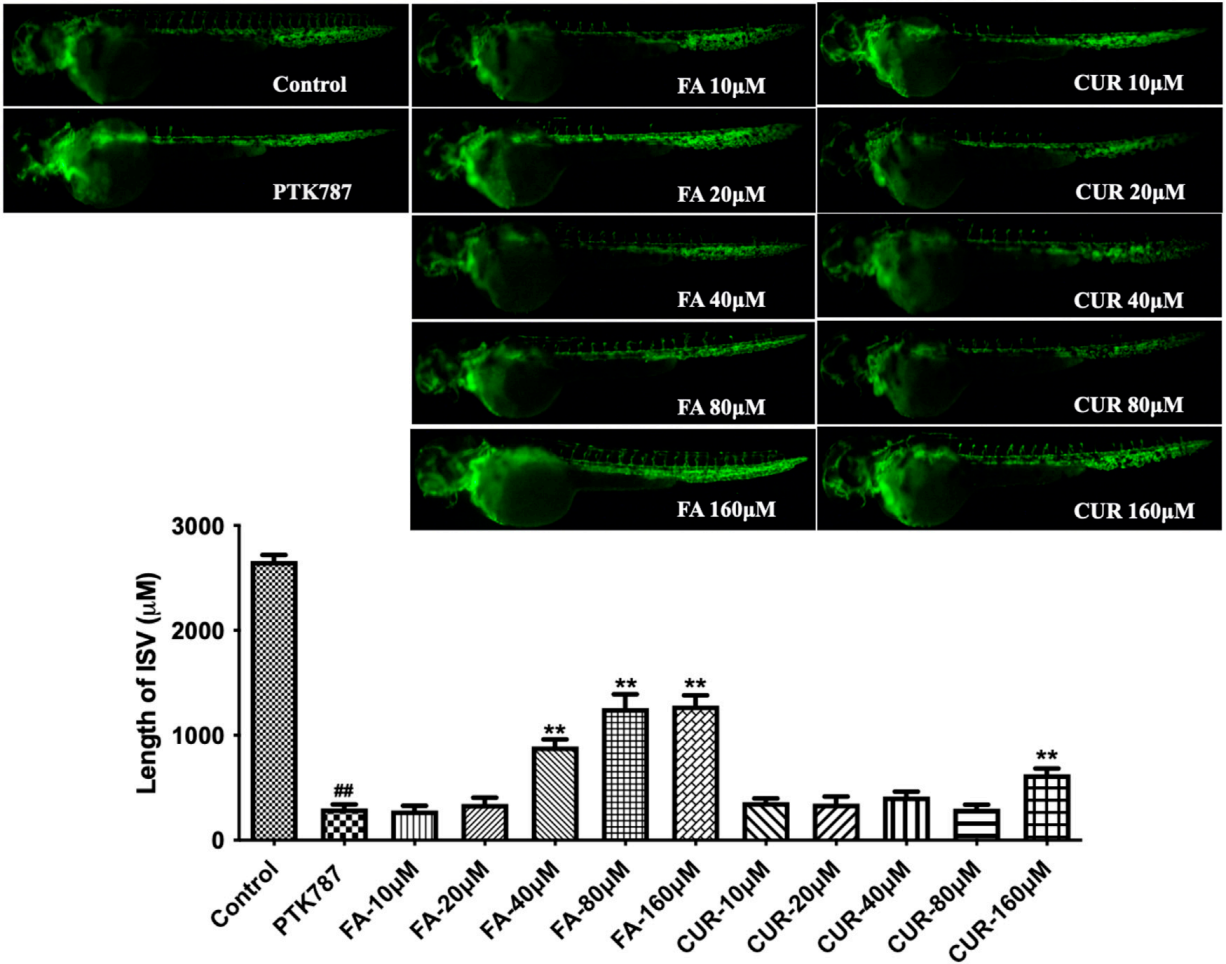
Ferulic acid (FA) and curculigostide (CUR) stimulated angiogenesis in a zebrafish model. Lateral view of zebrafish embryos treated with DMSO (0.1%, Control), PTK787 (0.2 μg/mL), PTK 787 plus FA (10, 20, 40, 80, and 160 μM) or CUR (10, 20, 40, 80, and 160 μM) for 24 h. FA (at 40, 80, and 160 μM) and CUR (at 160 μM) restored angiogenesis in ISVs against PTK787‐induced impairment. Statistical analysis data were shown as mean ± SEM (*n* ≥ 8). The comparison between groups was performed by student’s test. ^##^ *p* < 0.01 versus Control group; **p < 0.01 versus PTK787‐treated group.

Having established enriched prediction protein targets (Figure 2) and gene expression pathway signatures of the 51 known angiogenesis modulating individual metabolites (Figure 3), we next validated the angiogenesis-modulating activities of four specific individual metabolites using an *in vitro* endothelial tube formation assay (Bishop et al., 1999) and a zebrafish model of angiogenesis (Han et al., 2012). The individual metabolites selected were ferulic acid, curculigoside, deoxycholic acid and ursodeoxycholic acid. These were selected because of they have all been reported to have modulating activity in angiogenesis, including two promoters and two inhibitors. As shown in Figure 5, ferulic acid and curculigostide stimulated endothelial tube formation significantly compared to the control group at 100 nM (*p* < 0.05) and 1 µM (*p* < 0.05). And results in Figure 6 show that both ferulic acid and curculigostide stimulated angiogenesis in intersegmental vessels (ISVs) of zebrafish against PTK787-induced impairment, respectively. In more detail, ferulic acid significantly promoted angiogenesis in ISVs from 40 to 160 µM (*p* < 0.01) and curculigoside promoted angiogenesis in ISVs at 160 µM (*p* < 0.01). However, despite the reported anti-angiogenic and anti-tumour activities of a deoxycholic acid derivative (Alam et al., 2014) and anti-angiogenic activities of ursodeoxycholic acid and its derivatives in chick embryo chorioallantoic membrane (CAM) assay (Soma et al., 2006), Figure 7 shows these two bile acids had no effects on angiogenesis in the ISVs at any of the three doses tested (*p* > 0.05).

**FIGURE 7:**
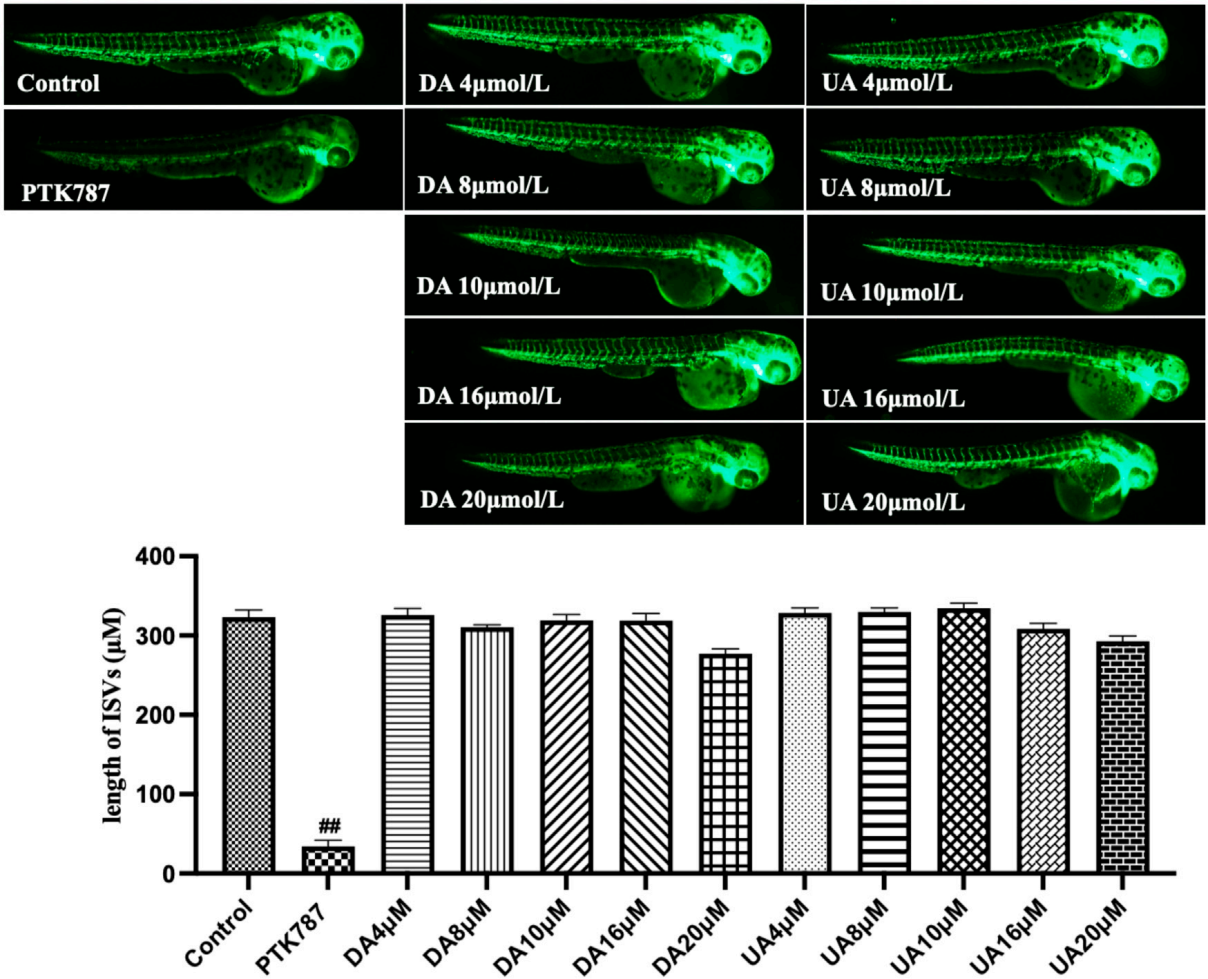
Lack of anti-angiogenic effects of deoxycholic acid (DA) and ursodeoxycholic acid (UA) in a zebrafish model. Lateral view of zebrafish embryos treated with DMSO (0.1%, Control), PTK787 (0.2 μg/mL), two bile acids added for 24 h. At 16 μM of each bile acids, some deformity was apparent, with the degree of malformation (yolk sac edema, tail malformation) increased at 20 μM. Compared with the Control group, these two bile acids had no effects on angiogenesis in the ISVs. In contrast, PTK787 effectively inhibited the growth of the ISVs. Statistical analysis data were shown as mean ± SEM (n ≥ 8). The comparison between groups was performed by student’s test. ^##^ *p* < 0.01 versus Control group.

### 3.5 Prediction of novel angiogenesis modulators by machine learning and biological assessment

Next, a Random Forest model was built to distinguish between promoting and inhibiting effects of individual metabolites with unknown angiogenic activity (*n* = 49 out of 100) with which to subsequently validate through *in-vitro* and zebrafish assays. The model was trained using the 51 individual metabolites with literature evidence of angiogenic inhibition (Supplementary Table S1). One reason for this is that we did not aim to perform a “virtual screen,” where just activity against a protein target, or process, is desired—but rather we aimed to differentiate between functional effects of individual metabolites, namely those promoting and those inhibiting angiogenesis, which is much more difficult to elucidate as it is a much more subtle aspect of compound action. The sensitivity and specificity of the model for promoters were found to be 0.74 and 0.62, respectively. The overall accuracy was 0.67 and the area under the receiver operating characteristic area under curve (ROC-AUC) was 0.64.

Table 2 ranks all the 49 individual metabolites with unknown angiogenesis-modulating activities according to their respective probability value (predictions ranged from 0.658 to 0.130) to promote or inhibit angiogenesis, with values close to 1 for promoters, and values close to 0 for inhibitors. From this list, we selected eight individual metabolites (stachydrine hydrochloride, hyperoside, tetrahydropalmatine, ginsenoside Rb3, ginsenoside Rc, 1-beta-hydroxyalantolactone, cinobufotalin and isoalantolactone) for biological assessment.

**TABLE 2:** Prediction results of 49 individual “unknown metabolites” ranking their probability of being pro-angiogenic or anti-angiogenic by Machine Learning developed in the current study. Prediction Gene means predicted by gene expression; Prediction TP means predicted by PIDGIN targets; Prediction Both means predicted by both gene expression and PIDGIN targets. The probability of individual TCM metabolites being pro-angiogenic or anti-angiogenic is ranked by Prediction Both, with values close to 1 for promoters, and values close to 0 for inhibitors.

	Compound name	Prediction both	Prediction gene	Prediction TP
1	Stachydrine hydrochloride	0.658	0.620	0.446
2	Aconitine	0.644	0.630	0.506
3	Anhydroicaritin	0.628	0.528	0.734
4	Ephedrine hydrochloride	0.576	0.590	0.504
5	Hyperoside	0.544	0.536	0.604
6	Gastrodin	0.48	0.498	0.396
7	Acteoside	0.462	0.442	0.604
8	Geniposide	0.458	0.418	0.436
9	Gallic acid	0.438	0.448	0.424
10	Lobetyolin	0.43	0.452	0.388
11	Cholic acid	0.406	0.402	0.404
12	Saikosaponin A	0.404	0.432	0.294
13	Tetrahydropalmatine	0.402	0.300	0.666
14	Hyodeoxycholic acid	0.396	0.398	0.372
15	Schisantherin A	0.384	0.280	0.728
16	Schizandrin	0.382	0.290	0.678
17	Benzyl benzoate	0.380	0.380	0.516
18	Ginsenoside Rb3	0.370	0.410	0.242
19	Ginsenoside Rc	0.370	0.358	0.210
20	Liquiritin	0.366	0.404	0.512
21	Bruceine D	0.340	0.374	0.328
22	Santonin	0.338	0.444	0.288
23	Ainsliadimer A	0.338	0.374	0.206
24	Imperatorin	0.320	0.348	0.424
25	Honokiol	0.304	0.314	0.304
26	Isoborneol	0.302	0.314	0.082
27	Sanguinarine	0.294	0.380	0.234
28	Japonicone A	0.282	0.336	0.116
29	L-scopolamine	0.274	0.334	0.262
30	beta-ecdysterone	0.268	0.298	0.298
31	Protocatechuic aldehyde	0.264	0.296	0.536
32	1 beta-hydroxyalantolactone	0.262	0.338	0.100
33	Gentiopicroside	0.258	0.330	0.418
34	Britanin	0.256	0.320	0.142
35	Salvianic acid A sodium	0.252	0.332	0.380
36	Sennoside A	0.248	0.382	0.612
37	Bacopaside I	0.240	0.324	0.248
38	Daidzin	0.224	0.350	0.594
39	Benzoylhypaconitine	0.224	0.334	0.300
40	Macrozamin	0.220	0.390	0.368
41	Benzoylaconitine	0.214	0.290	0.372
42	Phillyrin	0.192	0.238	0.366
43	(+) 2-(1-hydroxyl-4- oxocyclohexyl) ethyl caffeate	0.186	0.38	0.398
44	Hypaconitine	0.172	0.316	0.150
45	Cinobufotalin	0.162	0.370	0.230
46	Telocinobufagin	0.16	0.398	0.166
47	Bufotaline	0.154	0.362	0.110
48	Resibufogenin	0.132	0.294	0.166
49	Isoalantolactone	0.130	0.284	0.078

Firstly, stachydrine hydrochloride and hyperoside were tested using the *in vitro* endothelial tube formation assay because the probabilities obtained from the Prediction Gene model (0.620 and 0.536) and Prediction TP model (0.446 and 0.604) indicate that they are more likely to protome angiogenesis. However, they are both produced no effect on endothelial tube formation.

**FIGURE 8:**
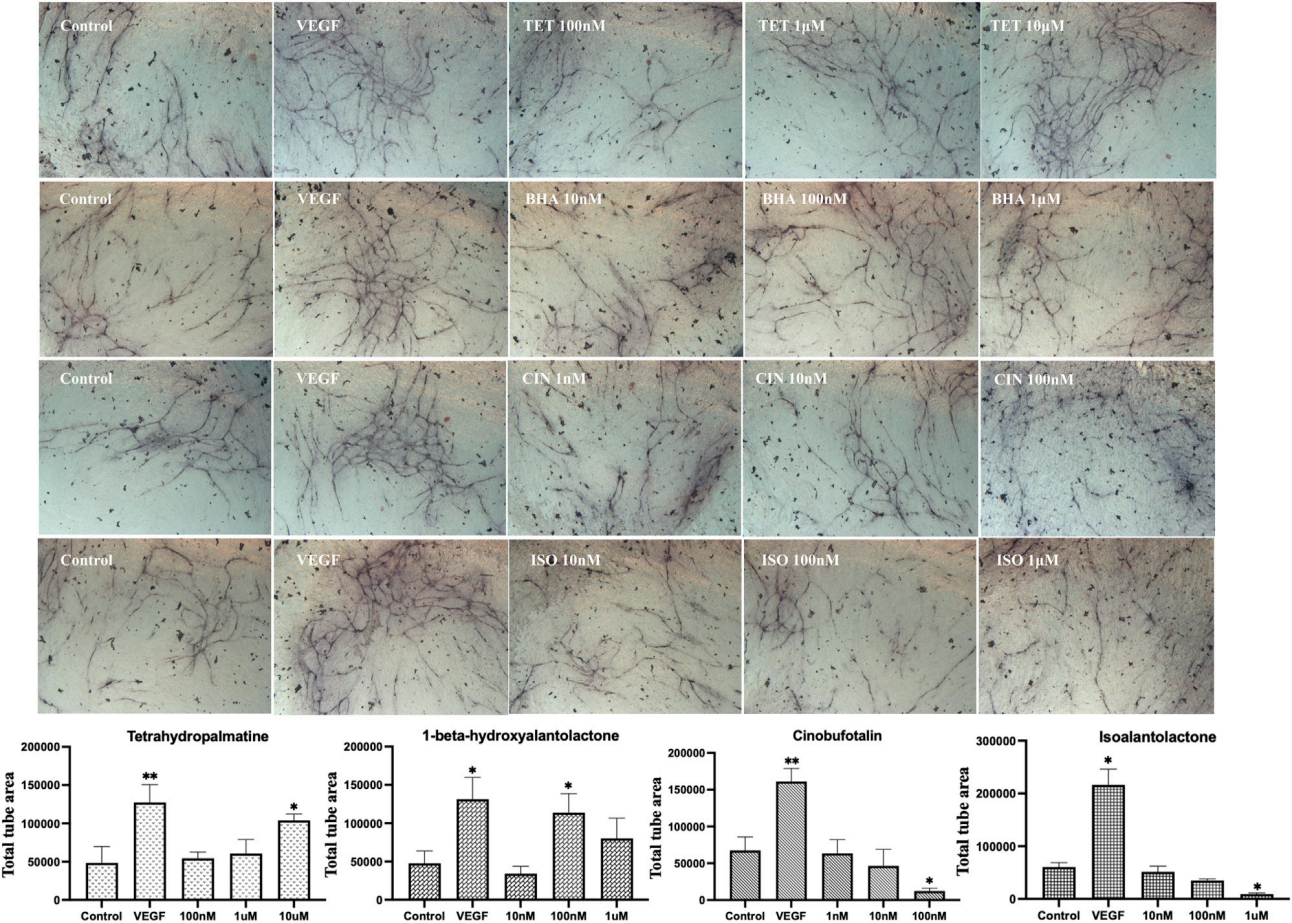
Modulation of endothelial tube formation *in vitro* by tetrahydropalmatine (TET), 1 beta-hydroxyalantolactone (BHA), cinobufotalin (CIN) and isoalantolactone (ISO) selected by the Machine Learning model. Total tube area was shown. Statistical analyses were performed by one‐way ANOVA followed by Dunnett’s *post hoc* test. Data represents mean ± SEM (*n* = 4–6). Compared with the control group, ^*^ *p* < 0.05, ^**^ *p* < 0.01, ^***^ *p* < 0.001.

**FIGURE 9:**
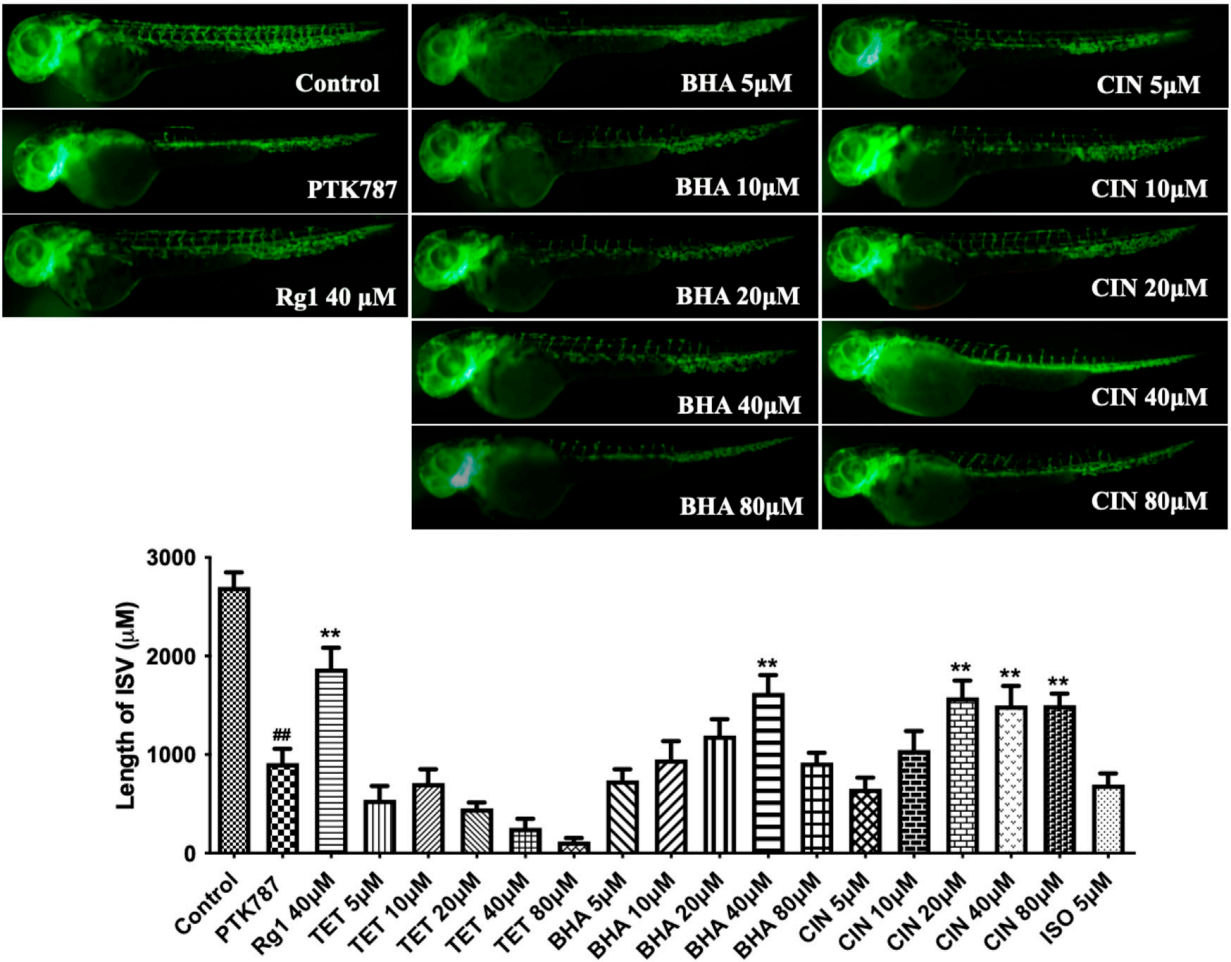
1-beta-hydroxyalantolactone (BHA) and Cinobufotalin (CIN) stimulated angiogenesis in a zebrafish model. Lateral view of zebrafish embryos treated with DMSO (0.1%, Control), PTK787 (0.2 μg/mL), PTK 787 plus ginsenoside Rg1 (Rg1 40 μM, positive control), or PTK 787 plus the test individual metabolites from TCM (5, 10, 20, 40, and 80 μM) for 24 h. 1-beta-hydroxyalantolactone (BHA) and cinobufotalin (CIN) dose-dependently restored angiogenesis in ISVs against PTK787‐induced impairment. Tetrahydeopalmatine (TET) worsened the effect of PTK787 and isoalantolactone (ISO) caused lethality at 10 μM and higher concentrations. Quantification of total length of ISVs in different groups from three independent experiments. Statistical analysis data were shown as mean ± SEM (*n* ≥ 8). The comparison between groups was performed by student’s test. ^##^ *p* < 0.01 versus Control group; **p < 0.01 versus PTK787‐treated group.

Next results shown in Figure 8 confirmed that tetrahydropalmatine (*p* < 0.05) at 10 μM and 1-beta-hydroxyalantolactone (*p* < 0.05) at 100 nM stimulated endothelial tube formation, while cinobufotalin (*p* < 0.05) at 100 nM and isoalantolactone (*p* < 0.05) at 1 μM inhibited endothelial tube formation. Therefore, further experiments were performed to determine the angiogenic effect of these four individual metabolites *in vivo*. The results in Figure 9 show that 1-beta-hydroxyalantolactone (*p* < 0.01) at 40 μM and cinobufotalin (*p* < 0.01) at 20, 40, and 80 μM restored angiogenesis in ISVs against PTK787‐induced impairment. Tetrahydropalmatine worsened the effect of PTK787. Isoalantolactone caused lethality at 10 μM and higher concentrations Intriguingly, cinobufotalin showed contradictory angiogenic activities *in vitro* and *in vivo* (Figure 8 vs. Figure 9). It is possible that the *in vivo* experimental results using the simple zebrafish angiogenesis model in this study differ from those observed using other animal models (e.g., chicken embryo chorionic villus experiment, tumour-bearing mice), and these are worthy of further investigation in the future.

**FIGURE 10:**
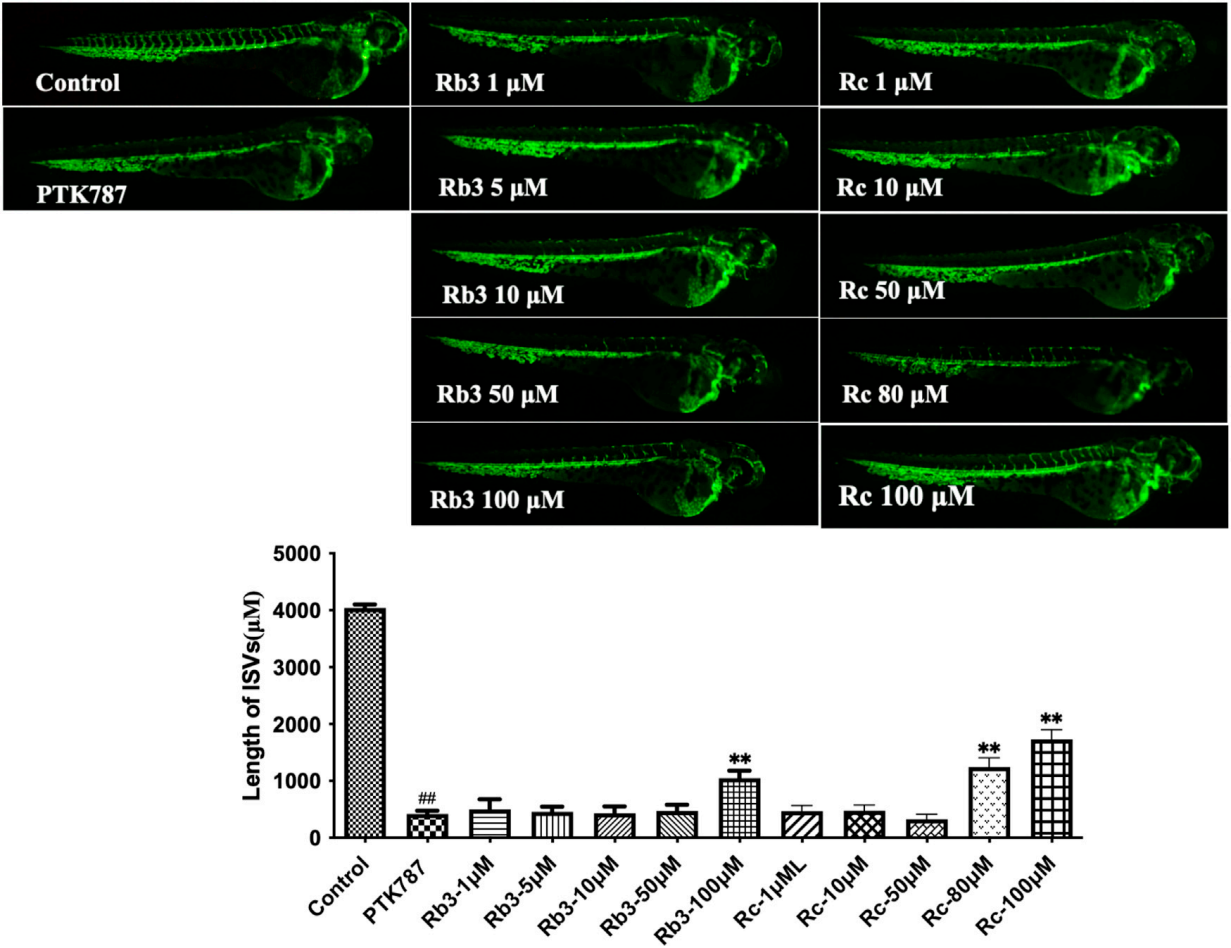
Ginsenosides Rb3 and Rc stimulated angiogenesis in a zebrafish model. Lateral view of zebrafish embryos treated with DMSO (0.1%, Control), PTK787 (0.2 μg/mL), PTK 787 plus ginsenoside Rb3 (1, 5, 10, 50, and 100 μg/mL) or Rc (1, 10, 50, 80, and 100 μg/mL) for 24 h Rb3 (100 μg/mL) and Rc (80 and 100 μg/mL) restored angiogenesis in ISVs against PTK787‐induced impairment. Quantification of total length of ISVs in different groups from three independent experiments. Statistical analysis data were shown as mean ± SEM (*n* ≥ 4). The comparison between groups was performed by student’s test. ^##^ *p* < 0.01 versus Control group; **p < 0.01 versus PTK787‐treated group.

The relatively low probability scores of ginsenosides Rb3 and Rc from the Prediction Gene model (0.41 and 0.358) and Prediction TP model (0.242 and 0.210) indicate that they are more likely to inhibit angiogenesis than to stimulate it. Contrary to the model predictions, both individual metabolites stimulated angiogenesis in zebrafish model, overcoming the inhibitory effect of PTK787 (Figure 10). Hence, these data emphasised the importance of validating predictions by machine learning algorithms using biological experiments, as well as highlighting the possible discrepancies in outcome of such experiments due to non-identical *in vitro* and *in vivo* models used in biological assessment.

## 4 Discussion

This study represents a novel composite approach to analyse the mode of action (MoA) of 51 pro- and antiangiogenic individual metabolites derived from TCM, based on the most important ten predicted protein targets, Differentially Expressed Genes (DEGs) and pathway enrichment signatures, culminating in the creation of an machine learning model to prospectively predict the angiogenic function of novel individual metabolites. Prior examples of the concept exist (Iwata et al., 2017), for example (Ravindranath et al., 2015), analyzed compound structure, gene expression and protein target in parallel, and were able to show their complementarity for understanding compound MoA in some situations. Therefore, the use of protein targets and gene expression is important in the deconvolution of an MoA as it can provide a more in-depth understanding and holistic view of natural products.

### 4.1 Enrichment of target proteins and pathways associated with angiogenic activity

Analysis of the PIDGIN predicted targets and enriched pathways by clustering (Figures 2, 3) demonstrated that individual metabolites derived from TCM known to regulate angiogenesis are indeed able to modulate specific targets and signalling pathways with known importance in angiogenesis such as Galectin-3, Galectin-9, and P-selectin, as well as the TNF signalling pathway, Wnt signalling pathway and p53 signalling pathway. Therefore, the analysis of the enriched targets and signalling pathways contributes to the elucidation of the mechanisms by which these individual metabolites modulate the effects of angiogenesis, and also highlights a key area for future research where we can select individual metabolites enriched to specific targets for in-depth study. For example, the results suggest a high potential for ferulic acid to be enriched in P-selectin targets. Therefore, the MoA of ferulic acid in regulating angiogenesis specifically by acting on P-selectin targets and upstream and downstream signalling pathways can be investigated in depth.

### 4.2 Biological assessment of individual metabolites derived from TCM with known angiogenic activity

In addition to this, through *in vivo* and *in vitro* assessment of four selected individual metabolites derived from TCM known to have angiogenic modulating effects we were able to show that although these individual metabolites have been reported in the literature, they exhibit different activities due to the different pharmacological models used. For example, deoxycholic acid and ursodeoxycholic acid did not show angiogenic effects in ISVs or zebrafish *in vivo* despite being reported as angiogenic inhibitors in the literature. It is possible that the *in vivo* experimental results in this study using a simple zebrafish model of angiogenesis can differ from results observed using other animal models (e.g., chick embryo chorioallantoic membrane assay, tumour-bearing mice) reported in the literature. However, of the other two individual metabolites assessed we did report agreement between the prior literature and the *in vivo* and *in vitro* models. For example, ferulic acid and curculigoside stimulated endothelial tube formation and intersegmental vessels (ISVs) of zebrafish against PTK787-induced impairment.

Overall, the results found for deoxycholic acid and ursodeoxycholic acid signal caution is required when developing machine learning models based on literature evidence of inhibitory or pro-angiogenic individual metabolites. Uncertain designations resulting from contradictory *in vitro* and/or *in vivo* biological activities could detrimentally affect the predictive power of our first-generation machine learning model of angiogenesis modulation, leading to the prediction results not fully corroborating with the angiogenesis phenotype.

### 4.3 Biological assessment of prospective machine learning prediction of novel metabolite angiogenic activity

To understand the potential utility of machine learning to predict angiogenesis stimulation of inhibition, we developed a Random Forest model to predict and validate 49 TCM individual metabolites with unknown angiogenic activity. Due to the relatively small number of individual metabolites (*n* = 51) used in training the model, the accuracy and applicability of current machine learning protocol is expected to have certain applicability domain limitations. We then validated the prospective predictions of select individual metabolites using *in vitro* and *in vivo* assays. To our knowledge, this is the first time that ginsenosides Rb3 and Rc, cinobufotalin and 1-beta-hydroxyalantolacton which are present in TCM have been shown to promote angiogenesis in a zebrafish model. It is noteworthy that TCM in which the pro-angiogenic individual metabolites are present have historically been used to promote angiogenesis. For example, the Shexiang Baoxin pill which contains pro-angiogenic individual metabolites such as ginsenoside Rg1 (from *Panax ginseng* C.A.Mey [Araliaceae, Ginseng Radix et Rhizama]) and cinnamaldehyde (from *Cinnamomum cassia* Presl [Lauraceae, Cinnamomi Cortex]), is widely used for the treatment of stable angina pectoris, chest pain or discomfort caused by coronary heart disease in China (Hu et al., 2021). On the other hand, some TCM in which angiogenesis inhibitors are present are used to inhibit angiogenesis. In 1995 (Mochizuki et al., 1995), reported that both 20(R)- and 20(S)-ginsenoside-Rg3 possess an ability to inhibit the lung metastasis of tumour cells, and the mechanism of their anti-metastatic effect is related to inhibition of the adhesion and invasion of tumour cells, and also to anti-angiogenesis activity. The botanical drug Shenyi Jiaonang which contains high level of ginsenoside Rg3, has been used widely in China for the treatment of a variety of cancers.

Cinobufotalin is the primary and active component of Chan-Su, an aqueous extract from the parotoid glands and dried secretion (from *Bufobufo gargarizans* Cantor or *Bufo melanostictus* Schneider [Bufonidae, Bufonis Venenum]) widely used as a cardiotonic, diuretic, and hemostatic agent (Meng et al., 2021). Chan-Su peptides have been reported to have anti-angiogenic effect (Xia et al., 2019). In our study, cinobufotalin was found to inhibit endothelial tube formation *in vitro*, but promoted angiogenesis in zebrafish. Such contradictory findings suggest that this active ingredient still has many unknown pharmacological effects and should be further explored in depth.

Moreover, of interest is 1β-hydroxyalantolactone, (from *Inula japonica* Thunb or *Inula britannica* L. [Compositae, Inulae flos]), which has been reported in the literature to have alleviated the progression of pulmonary fibrosis effect (Yu et al., 2021). It is worth noting that no studies have been conducted on the role of *Inulae flos* in regulating angiogenesis, but pharmacological studies have shown anti-inflammatory, antitumor, antioxidant, antiallergy, antidiabetic, blood lipid reduction, skin whitening, liver protection, anticonstipation, and antinociceptive effects (Yang et al., 2021). Our results indicate a pro-angiogenic effect of 1β-hydroxyadamantane for the first time.

However, we did identify individual metabolites such as tetrahydropalmatine and isoalantolactone which were predicted to have angiogenic activity by the machine learning model but produced no inhibitory or pro-angiogenic effects *in vivo*. Hence, whether the discovery of the pro-angiogenic individual metabolites in this study can be translated to stimulate angiogenesis therapeutically (such as myocardial infarction or chronic wounds) remain to be confirmed by stringent biological verification in appropriate animal models of human disease and rigorous clinical trials.

Overall, the results indicate that, despite the limited data, the machine learning model in combination with gene expression analysis and predicted targets was able to predict the angiogenesis effects of individual metabolites. This is because it is often easier to distinguish no effect from either effect (so inactive vs. any modulator), than to classify functional effects (since similar targets are involved, just in different ways). Moreover, the approach presented here allows for the preliminary elucidation of individual metabolite’s MoA based on target proteins, differentially expressed genes, and the final prospective prediction of the machine learning model. In contrast to conducting a phenotypic screen, for example, using ISV and zebrafish assays which were shown to disagree in cases in the present study, this approach therefore can help advance general understanding of the biological mechanisms (target proteins, DEGs, and pathways) underlying compound angiogenic activity and inform future drug development of TCM metabolites. However, in future research, more in-depth studies need be carried out based on the prospective predictions of the machine learning model we developed with close attention to the choice of pharmacology models used to validate them.

## 5 Conclusion

We performed a systematic analysis to explore the molecular angiogenic mechanisms of 51 TCM metabolites using bioinformatics approaches and assessed the possible angiogenesis-modulating activities of 49 TCM metabolites by a Machine Learning model and pharmacological models. Through this analysis, we identified that many of the TCM components possess diverse MoAs, and this may explain the applications of TCM in treating various symptoms and diseases *via* angiogenesis. Future studies on the pharmacological mechanisms of modulation of angiogenesis by TCM should be investigated in depth when they are conducted. We also identified novel pro-angiogenic TCM metabolites for further research in the future. The machine learning approaches applied in this study could be easily expandable to elucidate molecular mechanisms of other TCM components and drugs.

## Data Availability

The datasets presented in this study can be found in online repositories. The names of the repository/repositories and accession number(s) can be found in the article/Supplementary Material.
