# Supplementary material for: In silico prediction and biological assessment of novel angiogenesis modulators from traditional Chinese medicine
Source: Front Pharmacol. 2023 Feb 3;14:1116081. doi: 10.3389/fphar.2023.1116081 (PMC9937659; doi:10.3389/fphar.2023.1116081)
Supplement: Supplementary file 1 [file DataSheet1.pdf]

SUPPLEMENTARY DATA

Table S1. Angiogenic profile of the 100 TCM compounds selected for this study.

Table S2. Ten most important target prediction features and ten differentially expressed genes (DEGs) were used in the decision tree model.

Figure S1. The angiogenesis phenotype of the 51 Known Angiogenesis Modulators in this study has been shown via a decision tree derived from the Top 10 PIDGIN prediction targets (TP).

Figure S2. The angiogenesis phenotype of the 51 Known Angiogenesis Modulators in this study has been shown via a decision tree derived from the Top 10 DEGs.

Table S1. Angiogenic profile of the 100 TCM compounds selected for this study.

	Name of compound	Effect on angiogenesis	ref		Name of compound	Effect on angiogenesis	ref
1	Glycyrrhizic acid	Inhibit	[1]	52	Anhydroicaritin	Unknown	
2	Ginkgolide B	Inhibit	[2]	53	Aconitine	Unknown	
3	Andrographolide	Inhibit	[3]	54	Stachydrine hydrochloride	Unknown	
4	Emodin	Inhibit	[4]	55	Ephedrine hydrochloride	Unknown	
5	Ginsenoside Rb1	Inhibit	[5]	56	Lobetyolin	Unknown	
6	Saikosaponin d	Inhibit	[6]	57	Saikosaponin A	Unknown	
7	Chlorogenic acid	Inhibit	[7]	58	Gastrodin	Unknown	
8	Artemisinin	Inhibit	[8]	59	Acteoside	Unknown	
9	Deoxycholic acid	Inhibit	[9]	60	Imperatorin	Unknown	
10	Ursodeoxycholic acid	Inhibit	[10]	61	Hyodeoxycholic acid	Unknown	
11	Ginsenoside Rb2	Inhibit	[11]	62	Cholic acid	Unknown	
12	Ginsenoside Re	Inhibit	[12]	63	Isoborneol	Unknown	
13	Ginsenoside Rd	Inhibit	[13]	64	Benzyl benzoate	Unknown	
14	Nitidine chloride	Inhibit	[14]	65	Ginsenoside Rb3	Unknown	
15	Alantolactone	Inhibit	[15]	66	Ginsenoside Rc	Unknown	
16	Bufalin	Inhibit	[16]	67	Protocatechuic aldehyde	Unknown	
17	Arenobufagin	Inhibit	[17]	68	Britanin	Unknown	
18	Cinobufagin	Inhibit	[18]	69	Japonicone A	Unknown	
19	Oxymatrine	Inhibit	[19]	70	Bacopaside I	Unknown	
20	Matrine	Inhibit	[20]	71	1β-hydroxyalantolactone	Unknown	
21	Osthole	Inhibit	[21]	72	Salvianic acid A sodium	Unknown	
22	Silybin	Inhibit	[22]	73	Isoalantolactone	Unknown	
23	Oleanic acid	Inhibit	[23]	74	Resibufogenin	Unknown	
24	Scutellarin	Inhibit	[24]	75	Telocinobufagin	Unknown	
25	Strychnine	Inhibit	[25]	76	Bufotaline	Unknown	
26	Magnolol	Inhibit	[26]	77	Cinobufotalin	Unknown	
27	6-gingerol	Inhibit	[27]	78	Daidzin	Unknown	
28	Oridonin	Inhibit	[28]	79	Schisantherin A	Unknown	
29	Dioscin	Inhibit	[29]	80	Schizandrin	Unknown	
30	Narciclasine	Inhibit	[30]	81	Phillyrin	Unknown	
31	Borneol	Inhibit	[31]	82	β-ecdysterone	Unknown	
32	Chelerythrine	Inhibit	[32]	83	Honokiol	Unknown	
33	Paeoniflorin	Inhibit/promote	[33][34]	84	Geniposide	Unknown	
34	Ferulic acid	Inhibit/promote	[35][36]	85	Gallic acid	Unknown	

35	Resveratrol	Inhibit/promote	[37][38]	86	Liquiritin	Unknown	
36	Salidroside	Inhibit/promote	[39][40]	87	L-scopolamine	Unknown	
37	Hydroxysafflor yellow A	Inhibit/promote	[41][42]	88	Gentiopicroside	Unknown	
38	Hesperidin	Inhibit/promote	[43][44]	89	Benzoylhypaconitine	Unknown	
39	Berberine	Promote	[45][46]	90	Benzoylaconitine	Unknown	
40	Puerarin	Promote	[47]	91	Tetrahydropalmatine	Unknown	
41	Bilobalide	Promote	[48]	92	Hypaconitine	Unknown	
42	Tanshinone IIA	Promote	[49]	93	Macrozamin	Unknown	
43	Ginsenoside Rg1	Promote	[50]	94	Sennoside A	Unknown	
44	Astragaloside IV	Promote	[51]	95	(+) 2-(1-hydroxyl-4-oxocyclohexyl) ethyl caffeate	Unknown	
45	Salvianolic acid B	Promote	[52]	96	Bruceine D	Unknown	
46	Chenodeoxycholic acid	Promote	[53]	97	Santonin	Unknown	
47	Cinnamic acid	Promote	[54]	98	Ainsliadimer A	Unknown	
48	Cinnamaldehyde	Promote	[55]	99	Hyperoside	Unknown	
49	Muscone	Promote	[56]	100	Sanguinarine	Unknown	
50	Curculigoside	Promote	[57]				
51	Notoginsenoside R1	Promote	[58]				

Table S2. Ten most important target prediction features and ten differentially expressed genes (DEGs) were used in the decision tree model.

	Name	Importance
DEGs	PHD finger protein 3	0.20041
	transducin beta like 1 X-linked	0.16206
	GC-rich sequence DNA-binding factor 2	0.12933
	protein tyrosine phosphatase non-receptor type 12	0.12281
	UDP glucuronosyltransferase family 2 member B15	0.11795
	glutamine-fructose-6-phosphate transaminase 2	0.11473
	Fos proto-oncogene, AP-1 transcription factor subunit	0.07762
	activating transcription factor 6 beta	0.07510
	zyxin	0.0
	zymogen granule protein 16	0.0
Target Prediction	Ribosomal protein S6 kinase alpha 5	0.14535
	Endoplasmic reticulum aminopeptidase 1	0.10378
	Bile salt export pump	0.10233
	CaM-kinase kinase beta	0.09279
	Carbonic anhydrase III	0.08336
	Testis-specific serine/threonine-protein kinase 1	0.07195
	Sodium/glucose cotransporter 2	0.06999
	Mitogen-activated protein kinase kinase kinase 5	0.06545
	Maltase-glucoamylase	0.06431
	Tubulin 8	0.05494

Figure S1. The angiogenesis phenotype of the 51 Known Angiogenesis Modulators in this study has been shown via a decision tree derived from the Top 10 PIDGIN prediction targets (TP).

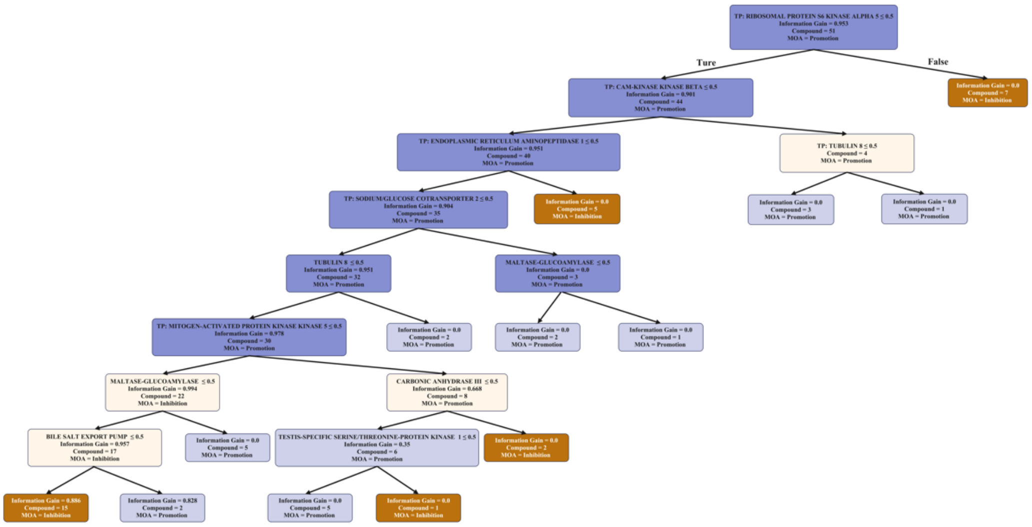

Figure S2. The angiogenesis phenotype of the 51 Known Angiogenesis Modulators in this study has been shown via a decision tree derived from the Top 10 DEGs.

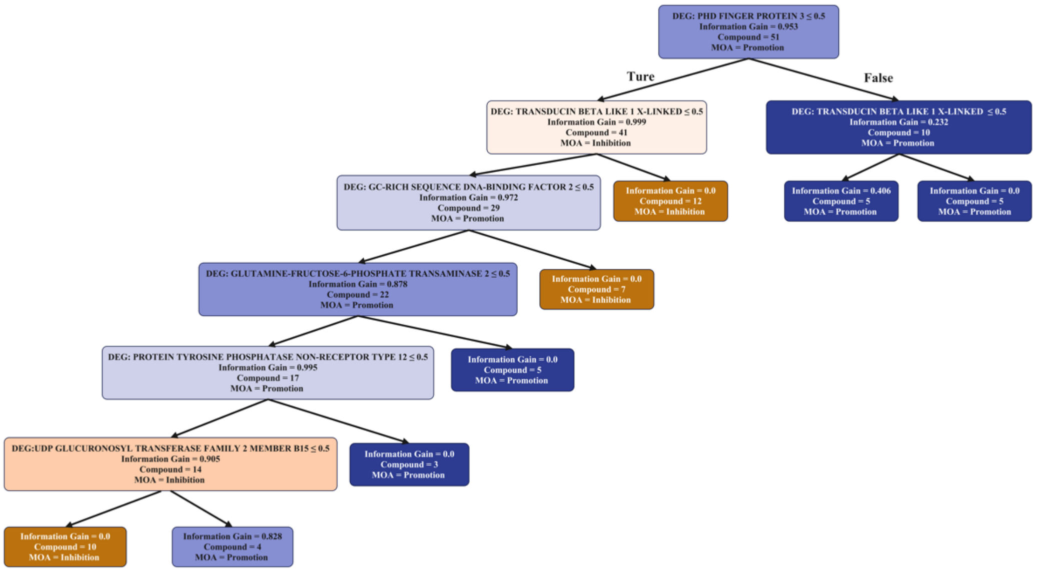

REFERENCES

[1] Kim KJ, Choi JS, Kim KW, Jeong JW. The anti-angiogenic activities of glycyrrhizic acid in tumor progression. Phytother Res. 2013,27(6):841-6. doi: 10.1002/ptr.4800.

[2] Sun L, He Z, Ke J, Li S, Wu X, Lian L, He X, He X, Hu J, Zou Y, Wu X, Lan P. PAF receptor antagonist Ginkgolide B inhibits tumourigenesis and angiogenesis in colitis-associated cancer. Int J Clin Exp Pathol. 2015,8(1):432-40.

[3] Dai J, Lin Y, Duan Y, Li Z, Zhou D, Chen W, Wang L, Zhang QQ. Andrographolide Inhibits Angiogenesis by Inhibiting the Mir-21-5p/TIMP3 Signaling Pathway. Int J Biol Sci. 2017,13(5):660-668. doi: 10.7150/ijbs.19194.

[4] Shi GH, Zhou L. Emodin suppresses angiogenesis and metastasis in anaplastic thyroid cancer by affecting TRAF6-mediated pathways in vivo and in vitro. Mol Med Rep. 2018,18(6):5191-5197. doi: 10.3892/mmr.2018.9510.

[5] Lu H, Zhou X, Kwok HH, Dong M, Liu Z, Poon PY, Luan X, Ngok-Shun Wong R. Ginsenoside-Rb1-Mediated Anti-angiogenesis via Regulating PEDF and miR-33a through the Activation of PPAR-γ Pathway. Front Pharmacol. 2017,8:783. doi: 10.3389/fphar.2017.00783.

[6] Wang BF, Cheng YA, Dang SS. [Angiogenesis inhibitory effect of saikosaponin-d on chicken embryo]. Zhongguo Zhong Xi Yi Jie He Za Zhi. 2009,29(5):425-429. Chinese.

[7] Zhong T, Piao L, Kim HJ, Liu X, Jiang S, Liu G. Chlorogenic Acid-Enriched Extract of Ilex kudingcha C.J. Tseng Inhibits Angiogenesis in Zebrafish. J Med Food. 2017,20(12):1160-1167. doi: 10.1089/jmf.2017.3987.

[8] Li Z, Ding X, Wu H, Liu C. Artemisinin inhibits angiogenesis by regulating p38 MAPK/CREB/TSP-1 signaling pathway in osteosarcoma. J Cell Biochem. 2019,11. doi: 10.1002/jcb.28424.

[9] Alam F, Al-Hilal TA, Chung SW, Seo D, Mahmud F, Kim HS, Kim SY, Byun Y. Oral delivery of a potent anti-angiogenic heparin conjugate by chemical conjugation and physical complexation using deoxycholic acid. Biomaterials. 2014,35(24):6543-6552. doi: 10.1016/j.biomaterials.2014.04.050.

[10] Suh H, Jung EJ, Kim TH, Lee HY, Park YH, Kim KW. Anti-angiogenic activity of ursodeoxycholic acid and its derivatives. Cancer Lett. 1997,113(1-2):117-122. doi: 10.1016/s0304-3835(97)04604-1.

[11] Sato K, Mochizuki M, Saiki I, Yoo YC, Samukawa K, Azuma I. Inhibition of tumor angiogenesis and metastasis by a saponin of Panax ginseng, ginsenoside-Rb2. Biol Pharm Bull. 1994,17(5):635-9. doi: 10.1248/bpb.17.635.

[12] Huang YC, Chen CT, Chen SC, Lai PH, Liang HC, Chang Y, Yu LC, Sung HW. A natural compound (ginsenoside Re) isolated from Panax ginseng as a novel angiogenic agent for tissue regeneration. Pharm Res. 2005,22(4):636-46. doi: 10.1007/s11095-005-2500-3.

[13] Zhang E, Shi H, Yang L, Wu X, Wang Z. Ginsenoside Rd regulates the Akt/mTOR/p70S6K signaling cascade and suppresses angiogenesis and breast tumor growth. Oncol Rep. 2017,38(1):359-367. doi: 10.3892/or.2017.5652.

[14] Chen J, Wang J, Lin L, He L, Wu Y, Zhang L, Yi Z, Chen Y, Pang X, Liu M. Inhibition of STAT3 signaling pathway by nitidine chloride suppressed the angiogenesis and growth of human gastric cancer. Mol Cancer Ther. 2012,11(2):277-287. doi: 10.1158/1535-7163.MCT-11-0648.

[15] Liu YR, Cai QY, Gao YG, Luan X, Guan YY, Lu Q, Sun P, Zhao M, Fang C. Alantolactone, a sesquiterpene lactone, inhibits breast cancer growth by antiangiogenic activity via blocking VEGFR2 signaling. Phytother Res. 2018,32(4):643-650. doi: 10.1002/ptr.6004.
[16] Wang H, Zhang C, Ning Z, Xu L, Zhu X, Meng Z. Bufalin enhances anti-angiogenic effect of sorafenib via AKT/VEGF signaling. Int J Oncol. 2016,48(3):1229-41. doi: 10.3892/ijo.2016.3326.
[17] Li M, Wu S, Liu Z, Zhang W, Xu J, Wang Y, Liu J, Zhang D, Tian H, Li Y, Ye W. Arenobufagin, a bufadienolide compound from toad venom, inhibits VEGF-mediated angiogenesis through suppression of VEGFR-2 signaling pathway. Biochem Pharmacol. 2012,83(9):1251-60. doi: 10.1016/j.bcp.2012.01.023.
[18] Li X, Chen C, Dai Y, Huang C, Han Q, Jing L, Ma Y, Xu Y, Liu Y, Zhao L, Wang J, Sun X, Yao X. Cinobufagin suppresses colorectal cancer angiogenesis by disrupting the endothelial mammalian target of rapamycin/hypoxia-inducible factor 1α axis. Cancer Sci. 2019,10(5):1724-1734. doi: 10.1111/cas.13988.
[19] Chen H, Zhang J, Luo J, Lai F, Wang Z, Tong H, Lu D, Bu H, Zhang R, Lin S. Antiangiogenic effects of oxymatrine on pancreatic cancer by inhibition of the NF-κB-mediated VEGF signaling pathway. Oncol Rep. 2013,30(2):589-95. doi: 10.3892/or.2013.2529.
[20] Han L, Zhang W, Li X, He Q, Han J, Zhang Y, Sun C, Zhou H, Liu K. Investigating the anti-angiogenic effects of Fufang Kushen Injection in combination with cisplatin using a zebrafish model. Pak J Pharm Sci. 2020,33(5):1955-1960.
[21] Yao F, Zhang L, Jiang G, Liu M, Liang G, Yuan Q. Osthole attenuates angiogenesis in an orthotopic mouse model of hepatocellular carcinoma via the downregulation of nuclear factor-κB and vascular endothelial growth factor. Oncol Lett. 2018,16(4):4471-4479. doi: 10.3892/ol.2018.9213.
[22] Deep G, Kumar R, Nambiar DK, Jain AK, Ramteke AM, Serkova NJ, Agarwal C, Agarwal R. Silibinin inhibits hypoxia-induced HIF-1α-mediated signaling, angiogenesis and lipogenesis in prostate cancer cells: In vitro evidence and in vivo functional imaging and metabolomics. Mol Carcinog. 2017,56(3):833-848. doi: 10.1002/mc.22537.
[23] Sogno I, Vannini N, Lorusso G, Cammarota R, Noonan DM, Albini A. Anti-angiogenic activity of a novel class of chemopreventive compounds, oleanic acid terpenoids. European Journal of Cancer Supplements.2008,6 (3):45. doi:org/10.1016/S1359-6349(08)70244-1.
[24] Long L, Li Y, Yu S, Li X, Hu Y, Long T, Wang L, Li W, Ye X, Ke Z, Xiao H. Scutellarin Prevents Angiogenesis in Diabetic Retinopathy by Downregulating VEGF/ERK/FAK/Src Pathway Signaling. J Diabetes Res. 2019:4875421. doi: 10.1155/2019/4875421. PMID: 31976335.
[25] Saraswati S, Agarwal SS. Strychnine inhibits inflammatory angiogenesis in mice via down regulation of VEGF, TNF-alpha and TGF-beta. Microvascular Research. 2013,87:7-13. doi:10.1016/j.mvr.2013.01.003.
[26] Kumar S, Guru SK, Pathania AS, Kumar A, Bhushan S, Malik F. Autophagy triggered by magnolol derivative negatively regulates angiogenesis. Cell Death Dis. 2013,4(10):e889. doi: 10.1038/cddis.2013.399.
[27] Eok-Cheon Kim, Jeong-Ki Min, Tae-Yoon Kim, Shin-Jeong Lee, Hyun-Ok Yang, Sanghwa Han, Young-Myeong Kim, Young-Guen Kwon,[6]-Gingerol, a pungent ingredient of ginger, inhibits angiogenesis in vitro and in vivo.Biochemical and Biophysical Research Communications.2005, 335(2): 300-308. doi.org/10.1016/j.bbrc.2005.07.076.

[28] Tian L, Xie K, Sheng D, Wan X, Zhu G. Antiangiogenic effects of oridonin. BMC Complement Altern Med. 2017,17(1):192. doi: 10.1186/s12906-017-1706-3.
[29] Zhou C, Li X, Zhou Z, Li C, Zhang Y. Comparative Transcriptome Analysis Identifies Genes Involved in Diosgenin Biosynthesis in Trigonella foenum-graecum L. Molecules. 2019,24(1):140. doi: 10.3390/molecules24010140.
[30] Jacqueline Bräutigam, Iris Bischoff, Christoph Schürmann, Giulia Buchmann, Jeremy Epah, Simone Fuchs, Elke Heiss, Ralf P. Brandes, Robert Fürst, Narciclasine inhibits angiogenic processes by activation of Rho kinase and by downregulation of the VEGF receptor 2. Journal of Molecular and Cellular Cardiology. 2019,135:97-108. doi:org/10.1016/j.yjmcc.2019.08.001.
[31] Li Y, Ren M, Wang J, Ma R, Chen H, Xie Q, Li H, Li J, Wang J. Progress in Borneol Intervention for Ischemic Stroke: A Systematic Review. Front Pharmacol. 2021,12:606682. doi: 10.3389/fphar.2021.606682.
[32] Jana J, Mondal S, Bhattacharjee P, Sengupta P, Roychowdhury T, Saha P, Kundu P, Chatterjee S. Chelerythrine down regulates expression of VEGFA, BCL2 and KRAS by arresting G-Quadruplex structures at their promoter regions. Sci Rep. 2017,7:40706. doi: 10.1038/srep40706.
[33] Abd El-Aal NF, Abdelbary EH. Paeoniflorin in experimental BALB/c mansoniasis: A novel anti-angiogenic therapy. Exp Parasitol. 2019,197:85-92. doi: 10.1016/j.exppara.2018.11.002.
[34] Xin QQ, Yang BR, Zhou HF, Wang Y, Yi BW, Cong WH, Lee SM, Chen KJ. Paeoniflorin Promotes Angiogenesis in A Vascular Insufficiency Model of Zebrafish in vivo and in Human Umbilical Vein Endothelial Cells in vitro. Chin J Integr Med. 2018,24(7):494-501. doi: 10.1007/s11655-016-2262-2.
[35] Yang GW, Jiang JS, Lu WQ. Ferulic Acid Exerts Anti-Angiogenic and Anti-Tumor Activity by Targeting Fibroblast Growth Factor Receptor 1-Mediated Angiogenesis. Int J Mol Sci. 2015,16(10):24011-31. doi: 10.3390/ijms161024011.
[36] Lin CM, Chiu JH, Wu IH, Wang BW, Pan CM, Chen YH. Ferulic acid augments angiogenesis via VEGF, PDGF and HIF-1 alpha. J Nutr Biochem. 2010 ,21(7):627-33. doi: 10.1016/j.jnutbio.2009.04.001.
[37] Wu H, He L, Shi J, Hou X, Zhang H, Zhang X, An Q, Fan F. Resveratrol inhibits VEGF-induced angiogenesis in human endothelial cells associated with suppression of aerobic glycolysis via modulation of PKM2 nuclear translocation. Clin Exp Pharmacol Physiol. 2018,45(12):1265-1273. doi: 10.1111/1440-1681.13017.
[38] Simão F, Pagnussat AS, Seo JH, Navaratna D, Leung W, Lok J, Guo S, Waeber C, Salbego CG, Lo EH. Pro-angiogenic effects of resveratrol in brain endothelial cells: nitric oxide-mediated regulation of vascular endothelial growth factor and metalloproteinases. J Cereb Blood Flow Metab. 2012,32(5):884-895. doi: 10.1038/jcbfm.2012.2.
[39] Kang DY, Sp N, Kim DH, Joung YH, Lee HG, Park YM, Yang YM. Salidroside inhibits migration, invasion and angiogenesis of MDA-MB 231 TNBC cells by regulating EGFR/Jak2/STAT3 signaling via MMP2. Int J Oncol. 2018,53(2):877-885. doi: 10.3892/ijo.2018.4430.
[40] Guo Q, Yang J, Chen Y, Jin X, Li Z, Wen X, Xia Q, Wang Y. Salidroside improves angiogenesis-osteogenesis coupling by regulating the HIF-1α/VEGF signalling pathway in the bone environment. Eur J Pharmacol. 2020,884:173394. doi: 10.1016/j.ejphar.2020.173394.

[41] Zhang J, Li J, Song H, Xiong Y, Liu D, Bai X. Hydroxysafflor yellow A suppresses angiogenesis of hepatocellular carcinoma through inhibition of p38 MAPK phosphorylation. Biomed Pharmacother. 2019,109:806-814. doi: 10.1016/j.biopha.2018.09.086.
[42] Zou J, Wang N, Liu M, Bai Y, Wang H, Liu K, Zhang H, Xiao X, Wang K. Nucleolin mediated pro-angiogenic role of Hydroxysafflor Yellow A in ischaemic cardiac dysfunction: Post-transcriptional regulation of VEGF-A and MMP-9. J Cell Mol Med. 2018,22(5):2692-2705. doi: 10.1111/jcmm.13552.
[43] Li W, Kandhare AD, Mukherjee AA, Bodhankar SL. Hesperidin, a plant flavonoid accelerated the cutaneous wound healing in streptozotocin-induced diabetic rats: Role of TGF-ß/Smads and Ang-1/Tie-2 signaling pathways. EXCLI J. 2018,17:399-419. doi: 10.17179/excli2018-1036.
[44] Kumar B, Gupta SK, Srinivasan BP, Nag TC, Srivastava S, Saxena R. Hesperetin ameliorates hyperglycemia induced retinal vasculopathy via anti-angiogenic effects in experimental diabetic rats. Vascul Pharmacol. 2012,57(5-6):201-7. doi: 10.1016/j.vph.2012.02.007.
[45] Och A, Podgórski R, Nowak R. Biological Activity of Berberine-A Summary Update. Toxins (Basel). 2020,12(11):713. doi: 10.3390/toxins12110713.
[46] Banaei P, Nazem F, Nazari A, Arjomand A. Preconditioning Effect of High-Intensity Interval Training (HIIT) and Berberine Supplementation on the Gene Expression of Angiogenesis Regulators and Caspase-3 Protein in the Rats with Myocardial Ischemia-Reperfusion (IR) Injury. Biomed Res Int. 2020:4104965. doi: 10.1155/2020/4104965.
[47] Ai F, Chen M, Yu B, Yang Y, Xu G, Gui F, Liu Z, Bai X, Chen Z. Puerarin accelerate scardiac angiogenesis and improves cardiac function of myocardial infarction by upregulating VEGFA, Ang-1 and Ang-2 in rats. Int J Clin Exp Med. 2015,8(11):20821-8.
[48] Zheng Y, Wu Z, Yi F, Orange M, Yao M, Yang B, Liu J, Zhu H. By Activating Akt/eNOS Bilobalide B Inhibits Autophagy and Promotes Angiogenesis Following Focal Cerebral Ischemia Reperfusion. Cell Physiol Biochem. 2018;47(2):604-616. doi: 10.1159/000490016.
[49] Li Z, Zhang S, Cao L, Li W, Ye YC, Shi ZX, Wang ZR, Sun LX, Wang JW, Jia LT, Wang W. Tanshinone IIA and Astragaloside IV promote the angiogenesis of mesenchymal stem cell-derived endothelial cell-like cells via upregulation of Cx37, Cx40 and Cx43. Exp Ther Med. 2018,15(2):1847-1854. doi: 10.3892/etm.2017.5636.
[50] Leung KW, Ng HM, Tang MK, Wong CC, Wong RN, Wong AS. Ginsenoside-Rg1 mediates a hypoxia-independent upregulation of hypoxia-inducible factor-1α to promote angiogenesis. Angiogenesis. 2011,14(4):515-22. doi: 10.1007/s10456-011-9235-z.
[51] Lin R, Chen H, Callow D, Li S, Wang L, Li S, Chen L, Ding J, Gao W, Xu H, Kong J, Zhou K. Multifaceted effects of astragaloside IV on promotion of random pattern skin flap survival in rats. Am J Transl Res. 2017,9(9):4161-4172.
[52] Lin S, Cui L, Chen G, Huang J, Yang Y, Zou K, Lai Y, Wang X, Zou L, Wu T, Cheng JCY, Li G, Wei B, Lee WYW. PLGA/β-TCP composite scaffold incorporating salvianolic acid B promotes bone fusion by angiogenesis and osteogenesis in a rat spinal fusion model. Biomaterials. 2019,196:109-121. doi: 10.1016/j.biomaterials.2018.04.004.
[53] Soma T, Kaganoi J, Kawabe A, Kondo K, Tsunoda S, Imamura M, Shimada Y. Chenodeoxycholic acid stimulates the progression of human esophageal cancer cells: A possible mechanism of angiogenesis in patients with esophageal cancer. Int J Cancer. 2006,119(4):771-782. doi: 10.1002/ijc.21917.

[54] Choi DY, Baek YH, Huh JE, Ko JM, Woo H, Lee JD, Park DS. Stimulatory effect of Cinnamomum cassia and cinnamic acid on angiogenesis through up-regulation of VEGF and Flk-1/KDR expression. Int Immunopharmacol. 2009,9(7-8):959-967. doi: 10.1016/j.intimp.2009.04.001.
[55] Yuan X, Han L, Fu P, Zeng H, Lv C, Chang W, Runyon RS, Ishii M, Han L, Liu K, Fan T, Zhang W, Liu R. Cinnamaldehyde accelerates wound healing by promoting angiogenesis via up-regulation of PI3K and MAPK signaling pathways. Lab Invest. 2018,98(6):783-798. doi: 10.1038/s41374-018-0025-8.
[56] Du Y, Ge Y, Xu Z, Aa N, Gu X, Meng H, Lin Z, Zhu D, Shi J, Zhuang R, Wu X, Wang X, Yang Z. Hypoxia-Inducible Factor 1 alpha (HIF-1α)/Vascular Endothelial Growth Factor (VEGF) Pathway Participates in Angiogenesis of Myocardial Infarction in Muscone-Treated Mice: Preliminary Study. Med Sci Monit. 2018,24:8870-8877. doi: 10.12659/MSM.912051.
[57] Kang Z, Zhu H, Luan H, Han F, Jiang W. Curculigoside A induces angiogenesis through VCAM-1/Egr-3/CREB/VEGF signaling pathway. Neuroscience. 2014,267:232-40. doi: 10.1016/j.neuroscience.2014.02.050.
[58] Zhong J, Lu W, Zhang J, Huang M, Lyu W, Ye G, Deng L, Chen M, Yao N, Li Y, Liu G, Liang Y, Fu J, Zhang D, Ye W. Notoginsenoside R1 activates the Ang2/Tie2 pathway to promote angiogenesis. Phytomedicine. 2020 ,78:153302. doi: 10.1016/j.phymed.2020.153302.
